# Real-time machine learning model to predict short-term mortality in critically ill patients: development and international validation

**DOI:** 10.1186/s13054-024-04866-7

**Published:** 2024-03-14

**Authors:** Leerang Lim, Ukdong Gim, Kyungjae Cho, Dongjoon Yoo, Ho Geol Ryu, Hyung-Chul Lee

**Affiliations:** 1 Department of Anesthesiology and Pain Medicine, Seoul National University College of Medicine, Seoul National University Hospital, 101 Daehak-ro, Jongno-gu, Seoul, 03080 Republic of Korea; 2 VUNO, 479 Gangnam-Daero, Seocho-gu, Seoul, 06541 Republic of Korea; 3 Department of Critical Care Medicine and Emergency Medicine, Inha University College of Medicine, 100 Inha-ro, Michuhol-gu, Incheon, 22212 Republic of Korea; 4 Department of Critical Care Medicine, Seoul National University College of Medicine, Seoul National University Hospital, 101 Daehak-ro, Jongno-gu, Seoul, 03080 Republic of Korea

**Keywords:** Intensive care units, Machine learning, Mortality, Prediction model, Validation study

## Abstract

**Background:**

A real-time model for predicting short-term mortality in critically ill patients is needed to identify patients at imminent risk. However, the performance of the model needs to be validated in various clinical settings and ethnicities before its clinical application. In this study, we aim to develop an ensemble machine learning model using routinely measured clinical variables at a single academic institution in South Korea.

**Methods:**

We developed an ensemble model using deep learning and light gradient boosting machine models. Internal validation was performed using the last two years of the internal cohort dataset, collected from a single academic hospital in South Korea between 2007 and 2021. External validation was performed using the full Medical Information Mart for Intensive Care (MIMIC), eICU-Collaborative Research Database (eICU-CRD), and Amsterdam University Medical Center database (AmsterdamUMCdb) data. The area under the receiver operating characteristic curve (AUROC) was calculated and compared to that for the National Early Warning Score (NEWS).

**Results:**

The developed model (iMORS) demonstrated high predictive performance with an internal AUROC of 0.964 (95% confidence interval [CI] 0.963–0.965) and external AUROCs of 0.890 (95% CI 0.889–0.891) for MIMIC, 0.886 (95% CI 0.885–0.887) for eICU-CRD, and 0.870 (95% CI 0.868–0.873) for AmsterdamUMCdb. The model outperformed the NEWS with higher AUROCs in the internal and external validation (0.866 for the internal, 0.746 for MIMIC, 0.798 for eICU-CRD, and 0.819 for AmsterdamUMCdb; *p* < 0.001).

**Conclusions:**

Our real-time machine learning model to predict short-term mortality in critically ill patients showed excellent performance in both internal and external validations. This model could be a useful decision-support tool in the intensive care units to assist clinicians.

**Supplementary Information:**

The online version contains supplementary material available at 10.1186/s13054-024-04866-7.

## Background

During the last decade, several machine learning models have been introduced to predict outcomes in the intensive care unit (ICU) [1–3]. These models include a gradient boosting machine (GBM) model for in-hospital mortality [3], a recurrent neural network-based model for major complications, and a hybridized convolutional neural network and long short-term memory (LSTM) model for 3 to 14-day mortality [4]. Previous studies have reported the excellent performance of models, suggesting their potential use in clinical practice [5].

However, the real-time clinical performance of models remains unclear because most models were developed to predict mid- to long-term outcomes using the first 24 h of ICU admission [6–9]. In general, the ICU mortality rate peaks in the first 24 h and then declines with management in the ICU [10, 11]. Before applying the models to routine monitoring in clinical practice, the performance of the models should be validated.

Another challenge in applying machine learning models to real-world clinical practice is that performance can vary depending on the training data and clinical setting [12]. A recent review article reported that approximately half of the ICU mortality prediction models have not been externally validated [13]. Some variables used in previous models, such as insurance type and diagnosis codes, are not standardized across countries, making them very difficult to apply internationally [3]. Therefore, models using variables commonly measured in most clinical settings should be developed and validated in multinational cohorts to ensure good performance in other clinical settings.

Here, we aimed to develop a machine learning-based real-time prediction model for short-term (24 h) mortality risk in critically ill patients using only variables readily available from electronic health records in most clinical settings. We reduced the number of input parameters to avoid overfitting and developed an ensemble model that uses the collective results of many different model architectures. We then validated the model’s performance using international datasets from Asia, America, and Europe. We hypothesized that the performance of the real-time model for predicting short-term ICU mortality using a minimal set of common clinical variables and ensemble machine learning techniques would be well maintained in international validation.

## Methods

### Ethical approval

The study protocol was approved by the Institutional Review Board (IRB) of Seoul National University Hospital (SNUH), South Korea with the title “Development of a machine learning model to predict major complications in ICU-patient” on December 6, 2021 (IRB No. 2111–140-1275). The IRB waived the requirement for informed consent due to the minimal risk of the study. This study was conducted in accordance with the Helsinki Declaration of 1975. The Medical Information Mart for Intensive Care (MIMIC)-III is publicly available after IRB approval by the Beth Israel Deaconess Medical Center in Boston, MA, USA (2001-P-001699/14) and the Massachusetts Institute of Technology, MA, USA (0403000206) [14]. The eICU-Collaborative Research Database (eICU-CRD) is publicly available with appropriate IRB approval from 208 hospitals in the USA [15]. The Amsterdam University Medical Center database (AmsterdamUMCdb) is publicly available with the approval of the Amsterdam University Medical Center, the Dutch patient organization IC Connect, and the Dutch Foundation of Family and Patient-Centered Care.

### Study population

Four different cohorts were used in this study: The SNUH internal cohort, MIMIC-III, eICU-CRD, and AmsterdamUMCdb datasets. The SNUH internal cohort data was collected from the patients admitted to five different ICUs at SNUH: the medical ICU (MICU), surgical ICU (SICU), coronary care unit (CCU), cardiopulmonary ICU, and emergency ICU between May 2007 and October 2021. Relevant data were extracted from electronic health records using SUPREME 2.0, a clinical data warehouse of SNUH. The MIMIC-III, eICU-CRD, and AmsterdamUMCdb are open datasets of critically ill patients that can be freely accessed after credentialing. The MIMIC-III includes data from 53,423 patients admitted to the Beth Israel Deaconess Medical Center between 2001 and 2012. The eICU-CRD includes 200,859 stays from 139,367 patients admitted to 335 units at 208 hospitals in the USA between 2014 and 2015. The AmsterdamUMCdb includes 23,106 stays at the Amsterdam University Medical Center between 2003 and 2016 (Additional file 1: Fig. E1).

**Fig. 1:**
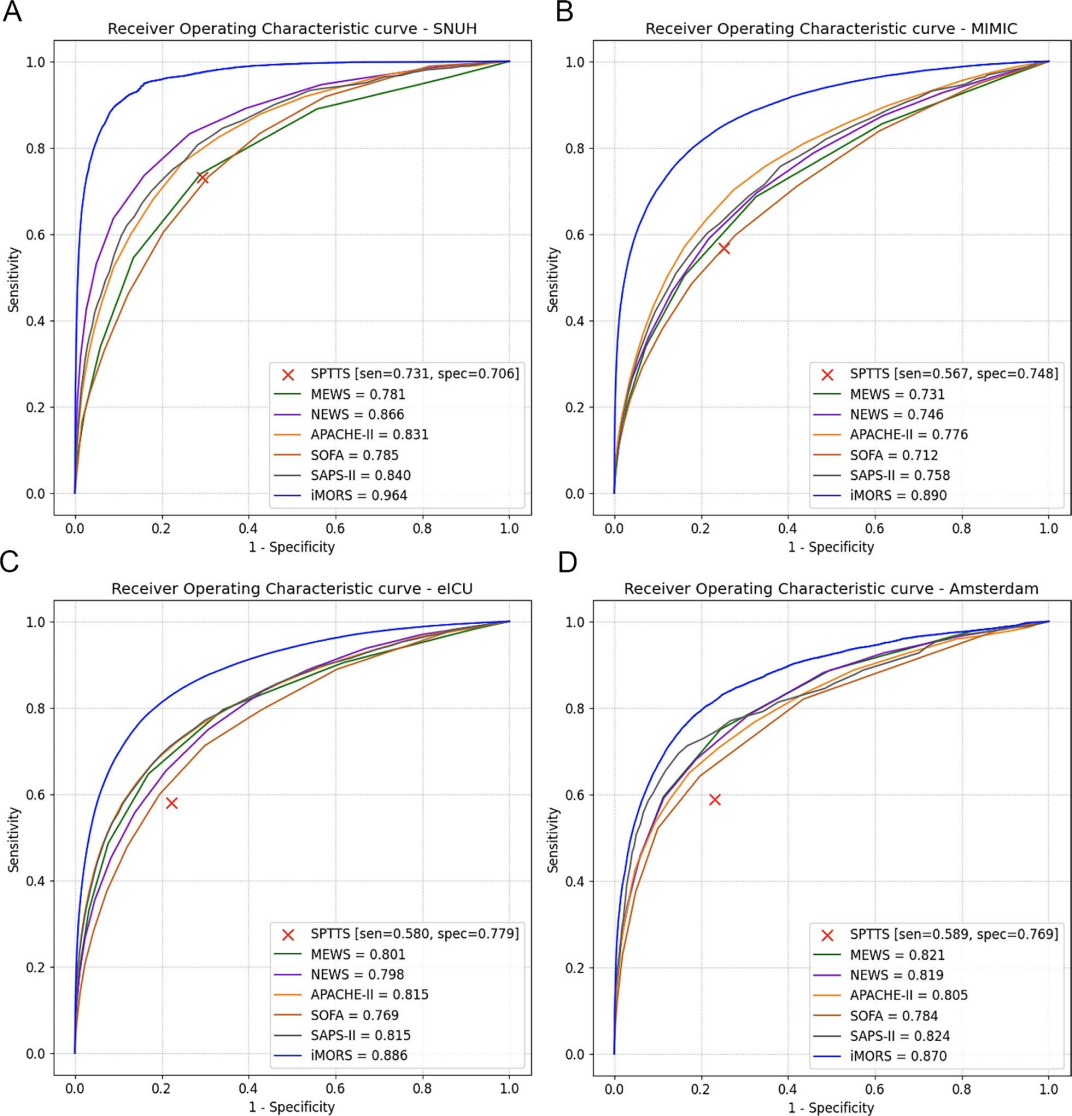
The area under the receiver operating characteristic curves for the cohorts. The label “iMORS” denotes our model, while the remaining models listed serve as comparisons. **A** internal validation on SNUH testing dataset **B** external validation on MIMIC **C** external validation on eICU-CRD (**D**) external validation on AmsterdamUMCdb. *SNUH S*eoul national university hospital, *MIMIC* Medical information mart for intensive care, *eICU-CRD* eICU collaborative research database, *AmsterdamUMCdb* Amsterdam university medical center database, *SPTTS* single-parameter weighted “track and trigger” systems, *NEWS* national early warning score, *MEWS* modified early warning score, *APACHE* acute physiology and chronic health evaluation, *SAPS* simplified acute physiology score, *SOFA* sequential organ failure assessment, *AUROC* area under the receiver operating characteristic, *sen* sensitivity, *spec* specificity

Patients younger than 18, refused life-sustaining treatment, had a “do not resuscitate” order, or had an ICU stay longer than 60 days were excluded.

### Data collection and preprocessing

An overview of the study process, including data collection, preprocessing, model development, and validation, is shown in Additional file 1: Fig. E2. A uniform preprocessing was employed across all the cohorts. Initially, diverse cohort datasets were combined into a single table, and similar features were grouped. Certain features underwent prioritization through manual assignment by clinical experts. For instance, invasive and non-invasive blood pressures were merged into a unified category, prioritizing invasive blood pressure when both values coexisted at the same time. For the other features, the average value was used as the representative value when multiple values were recorded concurrently.

**Fig. 2:**
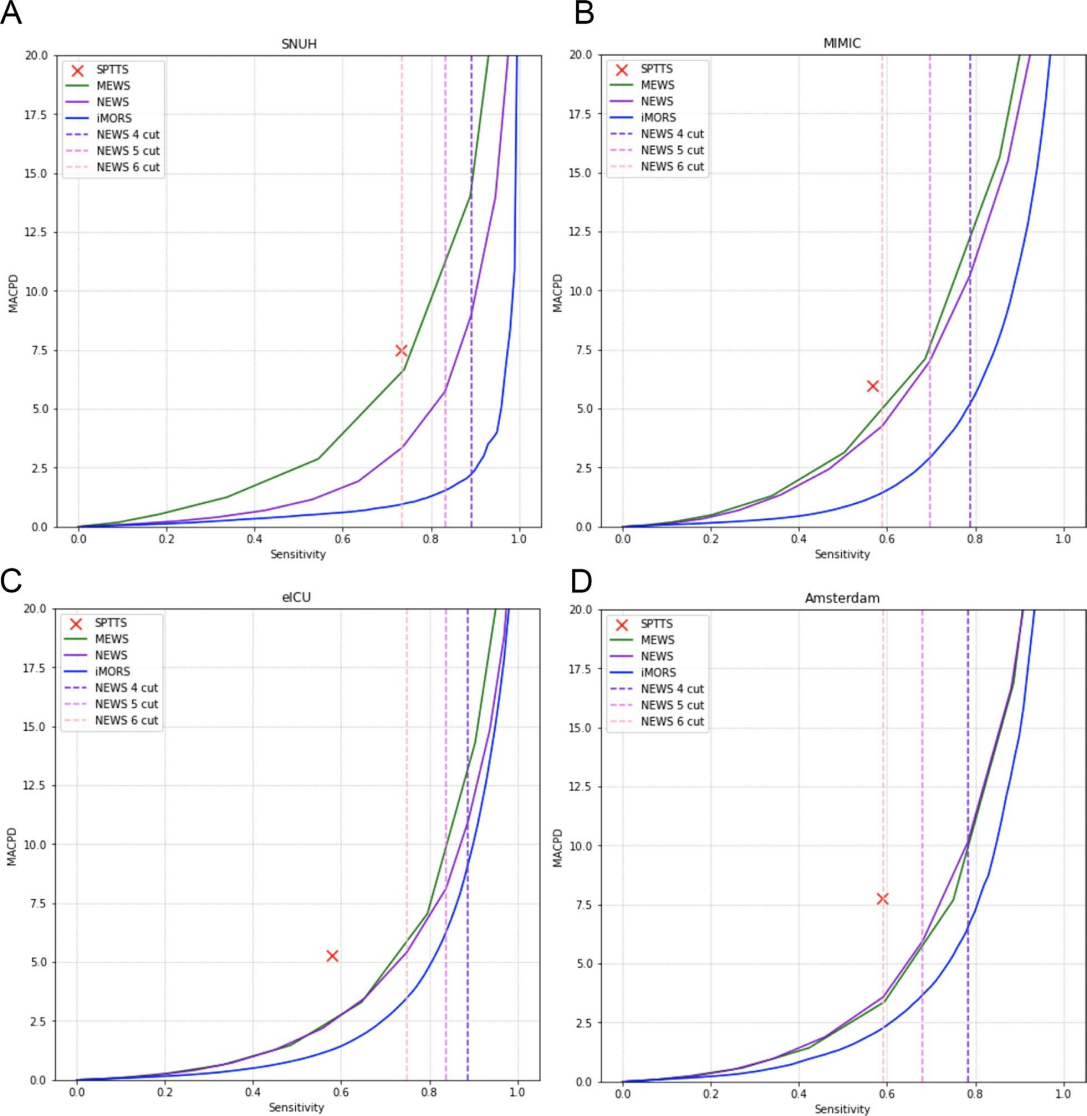
The mean alarm count per day (MACPD) for the study cohorts. MACPD is determined to indicate the average alarm count per bed every day. The MACPD values at NEWS 4, 5, and 6 cutoffs are compared with our model’s cutoff while maintaining the same sensitivity. **A** internal validation on SNUH testing dataset **B** external validation on MIMIC **C** external validation on eICU-CRD **D** external validation on AmsterdamUMCdb. *SNUH* Seoul national university hospital, *MIMIC* Medical information mart for intensive care, *eICU-CRD* eICU collaborative research database, *AmsterdamUMCdb* Amsterdam university medical center database, *SPTTS* single-parameter weighted “track and trigger” systems, *NEWS* national early warning score, *MEWS* modified early warning score

Samples were generated at each time point where at least one variable exists. Therefore, any variables not documented at that time point were treated as missing. Median imputation was applied when the initial value of the admission was missing. Otherwise, a forward-filling was applied to replace the missing values. Ultimately, standardization of feature values was conducted using statistical parameters derived from the training dataset.

A panel of clinical experts selected 30 candidate laboratory results readily collectable from most hospitals (Additional file 1: Table E1). Subsequently, occlusion analysis was performed using an LSTM-based model [16] with these variables [16]. This approach includes evaluating each variable’s impact on the model’s performance by setting the values of the variable to zero and assessing the model’s performance on the internal tuning dataset. By following the same process for all input features, we excluded the variable with the least decrease in the area under the receiver operating characteristic curve (AUROC) as it has the lowest impact on prediction performance. The process was iteratively repeated until an acceptable number of features were retained without a substantial drop in performance (decrease in AUROC < 0.002).

**Table 1:** Baseline characteristics of the study total cohorts

	Survival group				Mortality group			
	SNUH	MIMIC	eICU	Amsterdam	SNUH	MIMIC	eICU	Amsterdam
Number of admissions (%)	80,066 (99%)	42,501 (97%)	165,269 (98%)	13,279 (97%)	1,125 (1%)	1,524 (2%)	3,544 (2%)	354 (3%)
Number of samples (%)	9,026,225 (95%)	5,943,127 (99%)	15,847,502 (99%)	904,443 (98%)	113,799 (1%)	62,095 (1%)	149,646 (1%)	16,329 (2%)
Gender (female)	31,033 (39%)	17,894 (42%)	74,602 (45%)	4,442 (33%)	441 (39%)	662 (43%)	1,548 (44%)	121 (34%)
Age (year)	61.8 ± 14.6	62.1 ± 17.0	61.5 ± 16.9	59.4 ± 15.57	61.6 ± 15.7	67.1 ± 16.3	65.6 ± 16.2	63.34 ± 13.92
*9 vital signs*								
Respiratory rate (/min)	19.7 ± 6.4	19.9 ± 6.0	19.7 ± 5.9	18.09 ± 6.41	22.6 ± 7.8	22.2 ± 6.9	21.9 ± 6.71	22.58 ± 6.94
Heart rate (/min)	86.4 ± 19.8	86.1 ± 17.6	85.8 ± 18.4	81.82 ± 17.07	96.5 ± 24.5	91.5 ± 19.3	93.3 ± 21.1	92.15 ± 22.77
SBP (mmHg)	125.6 ± 24.7	123.5 ± 23.2	123.6 ± 23.5	126.89 ± 25.06	114.1 ± 27.5	113.3 ± 23.9	113.1 ± 25.1	116.64 ± 30.85
DBP (mmHg)	70.6 ± 13.7	61.6 ± 14.35	64.9 ± 14.9	62.91 ± 12.83	66.0 ± 16.1	57.3 ± 13.8	59.2 ± 15.0	60.64 ± 14.74
Body temperature (℃)	36.8 ± 0.7	37.1 ± 0.8	37.0 ± 0.7	36.71 ± 0.86	36.4 ± 1.1	36.9 ± 1.0	36.7 ± 1.3	36.09 ± 1.63
SpO2 (%)	98.2 ± 2.5	97.24 ± 2.7	96.8 ± 2.9	96.93 ± 2.88	95.9 ± 5.8	97.0 ± 3.9	96.2 ± 4.37	95.88 ± 4.21
GCS—eye	3.2 ± 1.1	3.51 ± 0.9	3.6 ± 0.8	3.41 ± 0.93	2.3 ± 1.3	2.8 ± 1.2	2.6 ± 1.3	1.65 ± 1.14
GCS—verbal	4.5 ± 1.0	4.58 ± 1.0	3.8 ± 1.7	4.48 ± 1.14	3.8 ± 1.4	3.7 ± 1.6	2.1 ± 1.6	2.17 ± 1.59
GCS—motor	5.2 ± 1.6	5.46 ± 1.2	5.7 ± 1.0	5.51 ± 1.23	3.6 ± 2.2	4.0 ± 2.0	4.0 ± 2.1	3.06 ± 2.22
*16 laboratory results*								
ALT (Units/L)	83.7 ± 220.0	187.0 ± 505.2	118.8 ± 377.6	99.05 ± 279.46	206.1 ± 488.4	313.5 ± 703.9	322.51 ± 718.2	239.26 ± 432.86
AST (Units/L)	90.5 ± 270.9	236.6 ± 759.6	159.0 ± 583.6	136.07 ± 435.77	271.9 ± 720.8	558.8 ± 1371.5	506.9 ± 1234.2	329.59 ± 439.05
Albumin (g/dL)	3.0 ± 0.5	2.9 ± 0.7	2.7 ± 0.7	2.4 ± 0.57	2.8 ± 0.5	2.7 ± 0.7	2.5 ± 0.7	2.21 ± 0.64
BUN (mg/dL)	25.3 ± 18.8	28.8 ± 22.6	27.2 ± 21.3	24.07 ± 18.28	36.2 ± 23.6	42.0 ± 27.2	39.6 ± 26.2	31.41 ± 21.99
Bilirubin (mg/dL)	2.0 ± 3.3	3.0 ± 5.3	1.5 ± 3.1	0.84 ± 1.36	6.1 ± 8.4	7.1 ± 9.9	3.6 ± 6.4	1.8 ± 3.36
CRP (mg/dL)	7.9 ± 7.6	9.9 ± 8.4	20.1 ± 40.0	8.54 ± 9.22	11.7 ± 8.5	12.4 ± 7.7	21.1 ± 44.5	10.97 ± 9.22
Chloride (mmol/L)	104.8 ± 6.4	104.9 ± 6.2	105.0 ± 7.2	107.77 ± 5.06	103.4 ± 8.2	104.5 ± 7.8	105.7 ± 7.9	106.5 ± 6.28
Creatinine (mg/dL)	1.3 ± 1.36	1.46 ± 1.49	1.48 ± 1.5	1.17 ± 1.16	1.59 ± 1.17	2.0 ± 1.6	2.0 ± 1.5	1.79 ± 1.15
Glucose (mg/dL)	161.6 ± 62.2	133.0 ± 46.8	147.4 ± 58.8	146.42 ± 42.21	164.1 ± 74.0	142.8 ± 64.5	152.6 ± 65.1	159.66 ± 71.51
Hemoglobin (g/dL)	10.5 ± 1.9	10.2 ± 1.7	10.3 ± 2.2	10.87 ± 1.76	9.7 ± 1.9	9.9 ± 1.8	10.1 ± 2.3	10.51 ± 1.92
Prothrombin time (INR)	1.3 ± 0.4	1.5 ± 0.7	1.6 ± 0.8	1.39 ± 0.4	1.7 ± 0.8	1.9 ± 1.0	1.9 ± 1.1	1.89 ± 0.96
Platelets (10^3^/µL)	167.1 ± 103.3	224.1 ± 138.0	203.5 ± 110.0	194.83 ± 111.73	105.7 ± 83.6	163.6 ± 131.8	150.3 ± 101.6	145.58 ± 129.52
Potassium (mmol/L)	4.0 ± 0.6	4.1 ± 0.62	4.0 ± 0.6	4.15 ± 0.47	4.1 ± 0.8	4.3 ± 0.8	4.3 ± 0.9	4.39 ± 0.82
Sodium (mmol/L)	138.0 ± 5.7	138.8 ± 5.1	139.0 ± 5.9	139.33 ± 4.43	139.0 ± 8.0	138.8 ± 6.7	140.8 ± 7.3	140.77 ± 6.19
WBC (10^3^/µL)	11.2 ± 5.9	11.7 ± 6.3	11.8 ± 6.4	13.26 ± 5.45	11.8 ± 8.6	13.83 ± 8.7	15.2 ± 10.2	14.49 ± 8.94
aPTT (sec)	39.4 ± 18.1	42.5 ± 23.9	45.6 ± 26.3	44.04 ± 18.43	52.74 ± 24.47	52.46 ± 29.5	51.9 ± 28.4	78.06 ± 62.36

The data is represented by the number of samples (%) or the mean and ± standard deviation. The percentage adds up to 100 for both the survival and mortality groups within each cohort. All *p*-values were < 0.001 except for the activated partial thromboplastin time in the mortality group (*p* = 0.062)

SNUH = Seoul National University Hospital; MIMIC = Mart for Intensive Care; eICU = eICU Collaborative Research Database; Amsterdam = Amsterdam University Medical Center database; SBP = systolic blood pressure; DBP = diastolic blood pressure; SpO2 = Saturation of peripheral oxygen; GCS = Glasgow coma scale; ALT = Alanine aminotransferase; AST = Aspartate aminotransferase; BUN = Blood urea nitrogen; CRP = C-reactive protein; INR = International normalized ratio; WBC = White blood cell; aPTT = Activated partial thromboplastin time

After feature selection, the final input comprised nine vital signs, 16 laboratory results, and age (Additional file 1: Table E2). Values outside predefined ranges were treated as missing data (refer to Additional file 1: Table E3). Besides these features, we included three time-delta features. The vital time-delta feature was defined as the time elapsed since the last measurement of any vital sign. The laboratory time-delta feature was defined as the time elapsed since the last measurement of any laboratory result. We also defined an ICU time-delta feature that measured the time elapsed since the ICU admission.

### Outcome definition

In the internal cohort, the mortality label was assigned to each admission based on the death certificates. In the other cohorts, we used the in-ICU death label of the dataset. For admissions with mortality labels, samples were extracted within 24 h before the death. Non-event samples were extracted from the entire period of the admissions without mortality labels.

### Model development

We trained several models with different architectures, including the transformer, light GBM, and LSTM-based deep learning (DL) models [16]. Particularly, we explored various LSTM-based models that integrated convolutional layers, fully connected layers, and LSTM layers. The final DL-based model structure was determined by exploring the performance of mortality prediction on the internal tuning dataset and comprised multiple feature-wise fully connected layers [17], three LSTM layers, and five fully connected layers with rectified linear unit activation [18].

Hyperparameter optimization was performed for the DL-based model via the grid search method. The specific search space for each hyperparameter and the selected values are provided in Additional file 1: Table E4. The light GBM model underwent optimization using the Tree-Structured Parzen Estimator, an element of the Optuna hyperparameter optimization framework [19]. Each candidate model underwent individual training, and diverse ensemble combinations were explored. Ultimately, the most proficient ensemble configuration, involving three light GBM models and a DL-based model, was identified as the final model. Ensemble integration entailed averaging the prediction scores of each model with equal weight. The transformer model was excluded due to relatively low performance.

Several regularization techniques were applied to prevent overfitting, including dropout, early stopping, contrastive loss as an auxiliary loss, and weight decay optimizers, such as AdamW and stochastic gradient descent. Specifically, we applied a stochastic weight-averaging technique to obtain more generalized optima [20]. To mitigate the class imbalance issue, we balanced the training data by under-sampling the non-event class. For each training epoch, the model was exposed to all event samples and randomly selected non-event samples, of which the number was the multiple of all event samples. The multiple ratio was one of the hyperparameters and optimized. This approach ensured that, over multiple epochs, most of the non-event samples underwent the training process. Additionally, post-processing temperature scaling was used to improve the expected calibration error (ECE).

### Performance evaluation and statistical analysis

We divided our internal cohort into development and test datasets. Model development was performed using only the development set (Additional file 1: Fig. E1). The development set consisted of a training and a tuning dataset, which were used to train the parameters and to find the optimal hyperparameters, respectively. The test dataset was used only for internal validation of the model performance.

We used the most recent data for the test dataset in the internal validation because we wanted to evaluate the model’s performance in a situation that reflects current clinical practice. For this reason, the training dataset included data from patients admitted between May 2007 and December 2018. The tuning dataset included data from patients admitted between January 2019 and December 2019. The testing dataset included all remaining data, including data from patients admitted between January 2020 and October 2021. The rationale for segmenting the cohorts by year stemmed from potential differences in the distribution of data across seasons and was intended to avoid bias due to seasonal trends. The delineated data split ensures the inclusion of at least one year of data for each dataset. However, the monthly mortality rate showed no specific patterns in Additional file 1: Fig. E3.

**Fig. 3:**
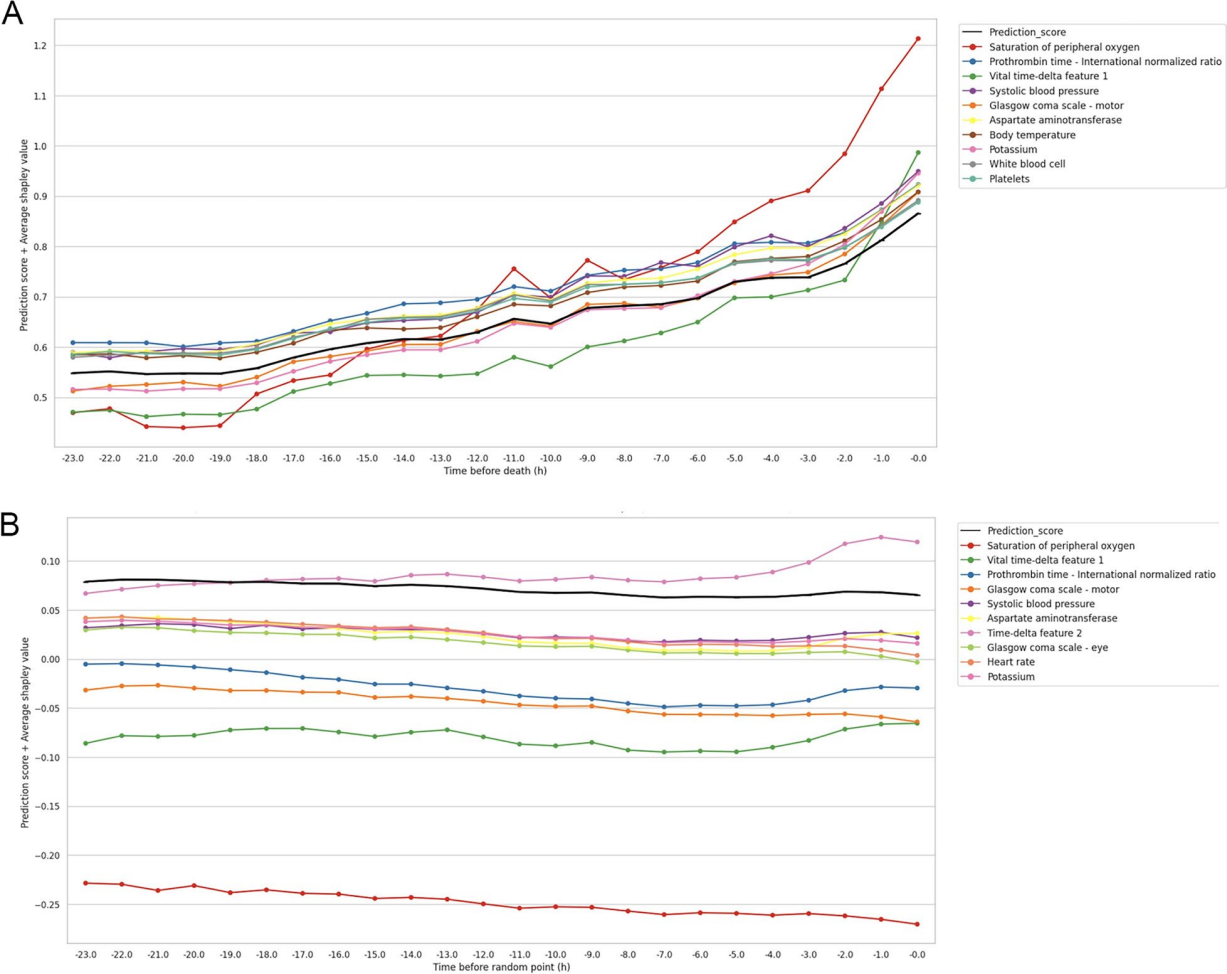
The average Shapley values in the internal testing dataset over time. The Shapley values are first averaged by hours for each admission and then averaged across all admissions. The black line represents the average prediction score. The average Shapley values are added to the prediction score in the figure. **A** For event admissions, we collected the values 24 h before death. **B** In the case of normal admissions, we gathered the values 24 h before random points to mitigate selective bias

The AUROC was used to compare the predictive performances of models. The model performance was compared with that of the single-parameter weighted track and trigger system (SPTTS) [18], Modified Early Warning Score (MEWS), National Early Warning Score (NEWS), Acute Physiology and Chronic Health Evaluation (APACHE)-II, Simplified Acute Physiology Score (SAPS), and Sequential Organ Failure Assessment (SOFA) scores. If these scores require time-varying data, we used the most recent data available. For example, we processed and incorporated real-time values, such as that for partial pressure of oxygen and alveolar-arterial oxygen difference, into the APACHE-II model.

The area under the precision-recall curves (AUPRC) was calculated as a secondary outcome. The calibration analysis was also performed, and the ECE was calculated to evaluate the models’ calibration. The mean alarm count per day (MACPD) was compared among the models at the same sensitivity level [21]. MACPD was calculated by dividing the total number of alarms during the patient’s ICU stay by the number of days in the ICU and then averaging these values across all ICU stays in the cohort. To determine the statistical significance of the feature values among the four cohorts, we calculated the *p*-value for each feature using the F-test.

### Feature importance

The Shapley values were calculated using randomly selected 300 patients in the test datasets. To ensure balance, a one-to-one sampling approach was employed, involving the selection of 150 events and 150 non-event admissions. We also calculated the change in feature importance over time to determine which variables are most important to the model’s output over time.

### Subgroup analysis

In the subgroup analysis, we categorized each cohort into four primary subgroups: ethnicity, ICU type, length of stay, and age. The ethnicities were stratified into six groups: African American, Asian, Caucasian, Hispanic, Native American, and Other/Unknown. ICU types were classified into four categories: MICU, SICU, CCU, and others. Comprehensive details regarding this classification are available in Additional file 1: Table E5 and Fig. E9. Age groups were discretized into intervals of 10 years, except for the 18 ~ 39 years age range. The length of stay was divided into 8-h intervals for the initial 24 h, followed by subsequent intervals of 1, 2, 4, and 8 days. The division of intervals for age and length of stay was conducted with reference to both the patient count and interval characteristics.

## Results

### Dataset construction

We included 307,907 of the 377,188 eligible ICU admissions from four cohorts in our study (Additional file 1: Table E5 and Fig. E1). In the model development phase, 70,644 non-event and 983 event ICU admissions were used, whereas 9,422 non-event and 137 event admissions were used for internal validation. In the external validation, we analyzed 42,501 non-event and 1,503 event admissions from the MIMIC-III [14], 165,421 non-event and 3,663 event admissions from the eICU-CRD [15], and 13,279 non-event and 354 event admissions from AmsterdamUMCdb (Additional file 1: Fig. E1). Baseline characteristics and mean and standard deviation of input features are shown in Table 1, according to the cohort and event group.

### Distribution discrepancy among cohorts

The NEWS was calculated based on vital signs, the alert, voice, pain, unresponsive scale, and the use of oxygen supply. NEWS values differed significantly among cohorts (Additional file 1: Fig. E4). Except for alanine aminotransferase, aspartate aminotransferase, and C-reactive protein levels, all input features differed significantly (*p*-values < 0.001) among cohorts (Additional file 1: Fig. E5).

### Predictive and alarm performance

Our model yielded an AUROC of 0.964 (95% confidence interval [CI], 0.963–0.965) in the internal testing dataset and 0.890 (95% CI, 0.889–0.891) in the MIMIC-III, 0.886 (95% CI, 0.885–0.887) in the eICU-CRD, and 0.870 (95% CI, 0.868–0.873) in the AmsterdamUMCdb. Notably, our model surpassed all other models in both internal and external validations, whereas all comparative models showed relatively similar performances (Fig. 1, and Additional file 1: Table E6, and Fig. E6).

In the calibration analysis, our model showed the lowest ECE in the internal and external cohorts, with an internal ECE of 0.146 and external ECEs of 0.205, 0.199, and 0.254 for the MIMIC-III, eICU-CRD, and AmsterdamUMCdb, respectively (Additional file 1: Fig. E8). In contrast, NEWS ranked second in terms of ECE, with an internal ECE of 0.259 and external ECEs of 0.249, 0.197, and 0.309 for the MIMIC, eICU-CRD, and AmsterdamUMCdb, respectively.

Regarding alarm performance, the MACPD of our model was 24–28% of that generated by NEWS (Fig. 2, and Additional file 1: Table E7) in the internal testing dataset. Notably, the MACPD in the external cohorts was still 33–83% of the alarms generated by the NEWS.

### Feature importance

The top five features included saturation of peripheral oxygen (SpO_2_), the vital time-delta feature, the Glasgow Coma Scale (GCS) motor score, systolic blood pressure, and the laboratory time-delta feature (Additional file 1: Fig. E7).

Figure 3 shows an analysis of feature importance over time. The plot illustrates the top 10 features based on the absolute sum of the Shapley values. The SpO_2_ value had a profound effect as the patients approached death. In contrast, in the survival group, most features contributed to decreased prediction scores over time. Among them, the contribution of the low SpO_2_ was significant.

### Subgroup analysis

Regarding ethnicity, our model showed similar performance trends across groups but with slight variations among the Asian (including Korean), Hispanic, and Native American groups. Notably, our model achieved the highest performance for the Native American group. In contrast, the performance for Asians was comparatively lower, even though the training dataset was constructed in the Asian population (Additional file 1: Fig. E9a).

For age and length of ICU stay, the performance trends were consistent across the external cohorts, with the AUROC decreasing as the length of ICU stay and age increased. This suggested that our model performed relatively better in predicting outcomes in patients with shorter stays and younger ages (Additional file 1: Fig. E9c, d). Regarding the ICU type, we grouped the different ICU types into four categories: MICU, SICU, CCU, and others. Detailed information on this classification is provided in (Additional file 1: Fig. E10). Our model showed significantly better performance for the MICU, except for the AmsterdamUMCdb, while achieving a lower performance for the CCU than for all ICUs in all cohorts (Additional file 1: Fig. E9b).

## Discussion

In this study, we developed and internationally validated a machine learning-based model for real-time mortality prediction within 24 h in critically ill patients. Although we developed our model based on single-center data, using common clinical features and ensemble techniques, our model outperformed conventional risk scores in the real-time application to the internal and external validation cohorts. However, the performance slightly declined in the external validation.

Previous studies have reported machine learning-based mortality prediction models for critically ill patients can achieve significantly better predictive performance than conventional scoring systems, such as the APACHE II or SAPS II [6, 9, 22–25]. However, most models were designed to predict mortality at a single time point such as 24 h after admission [22, 25], which hardly reflects management during the ICU stay. Real-time models have been only used during the first 24 h after admission [24], with a 1-day interval [23], or for long-term outcomes [6]. We developed a model that can be applied hourly, intended for real-time monitoring in the ICU, and evaluated its performance.

Previous studies have also reported that the accuracy of mortality prediction models declines in the later stages of the ICU stay [6, 26]. Despite exhibiting a similar decline in performance over time, our model’s AUROC remained above 0.82 in both internal and external validations, except for the AmsterdamUMCdb (Additional file 1: Fig. E9c). This could be interpreted as our model trained to predict short-term mortality and optimized for real-time performance. In both internal and external datasets, the score of our model consistently increased in the mortality cases as death approached, showing its utility for real-time monitoring in the ICU (Fig. 2a).

When applying real-time models in clinical practice, alarm fatigue is one of the major concerns that can lead to the complete inactivation of the alarms [27]. However, at a sensitivity level of 0.891, the MACPD of our model was 2.344, fewer than three alarms per bed per day. Although the alarm rate was increased more than twofold in the MIMIC-III, threefold in the AmsterdamUMCdb, and fourfold in the eICU-CRD, it was still significantly lower than that of the NEWS and was fewer than 10 alarms per bed per day. We considered this alarm rate acceptable and would not increase the risk of alarm fatigue.

Our model included features routinely measured and monitored in the ICU, such as heart rate, SpO_2_, or GCS, including commonly monitored variables that allow our model to be easily applied in daily care, without requiring specialized laboratory tests or monitoring equipment. Moreover, the model explained the predictions for each patient at each point in time. As shown in Additional file 1: Fig. E7, the Shapley values indicated the impact of each input feature on the model output. As the European Union’s General Data Protection Regulation took effect as a law in April 2018, the interpretability of the algorithmic decision-making model became essential [23]. Nevertheless, whether changing the variables based on feature importance improves the outcomes requires further investigation.

Although our model showed better calibration with the lowest ECE compared to other scoring systems, it still tended to underestimate the risk of mortality in both internal and external cohorts. The low mortality rate in the developmental cohort (1%) may be attributed to the underestimating model. Other models besides iMORS were initially developed to provide early warnings for the deterioration of patients in general wards and underestimate the risk of mortality [28]. Furthermore, although some models, such as APACHE II, were developed to suggest the risk of mortality for ICU patients, they provide an overall risk of mortality rather than short-term mortality. Considering that the calibration analysis of the cohort with the highest mortality rate, AmsterdamUMCdb, showed the highest ECE for all prediction models when compared to other datasets, we can speculate about the potential underestimation due to differences in mortality rate.

Regarding all subgroups of age, sex, ethnicity, insurance, and ICU type in both internal and external cohorts, our model showed good performances with an AUROC of > 0.85, suggesting the universal applicability of the prediction model for all types of critically ill patients. Interestingly, the AUROC of our model was highest for Native Americans in both the MIMIC-III and eICU-CRD and lowest for Asians in the eICU-CRD (Additional file 1: Fig. E8a). This divergence could be attributed to the limited number of Native American patients and possible differences in the data distribution of the Asian population in the eICU-CRD as compared to the Korean cohort used for training. Our model showed good predictive ability for all types of ICU, with AUROCs > 0.90 in the internal testing dataset and 0.83 in the external cohorts. In both internal testing dataset and external cohorts, the AUROC was consistently lower for the CCU than for the other ICU types except for AmsterdamUMCdb. This can be attributed to the distinct features of the CCU, which play a role in both the ICU and the post-procedural care unit after the cardiac intervention. At SNUH, the CCU plays a limited role as an ICU and does not provide specialized modalities, such as mechanical ventilators or continuous renal replacement therapy. Limited therapeutic options, such as the characteristics or severity of the illness, may affect the distribution of patients in the CCU.

The applicability of prediction models in clinical practice is as crucial as predictive performance. As our model utilized vital signs and laboratory tests routinely measured in the ICU, and the input design incorporated real-time updates with each new value, our model suggests the potential for an automated mortality prediction using real-time data from electronic health record systems. Furthermore, the model’s explainable nature, which identifies factors contributing to predicted mortality, indicates its potential utility as a clinical decision-support tool in clinical practice. Therefore, clinical trials that validate the clinical utility of the model are warranted. Additionally, improving the model’s performance by utilizing additional input, newer architecture, and more data should be considered in future studies.

This study has several limitations. First, we developed our model using data from a single tertiary academic hospital where the distribution of patients differed from that of other institutions. The presence of a specialized unit for close monitoring in the general ward, such as a “sub-ICU,” might imply an increased severity of illness in patients who are admitted to the ICU. Although the external validation using the MIMIC-III, eICU-CRD, and AmsterdamUMCdb showed that the model had good performance, the mortality rate was similar to or even higher in the external cohorts. Therefore, the prediction model should be applied with caution, and recalibration may be required for other cohorts, particularly those with lower mortality rates. Second, the predictive performance was reduced in the external cohort. As shown in Additional file 1: Table E2, there were differences in mortality among the cohorts, while the cohorts from the USA (MIMIC and eICU-CRD) are relatively similar. The difference in the severity of each feature may reduce the model’s performance. Third, the model’s performance on the external cohorts showed a decreasing trend as the ICU length of stay was prolonged and patients’ age increased. Except for the subgroup of the age 70–79 in the AmsterdamUMCdb, there was a consistent decline in the model’s predictive performance as patients’ ages increased and their ICU stay duration prolonged (Additional file 1: Fig. E9c, d). Despite a reduction in performance in high mortality risk subgroups, the model demonstrated an acceptable AUROC of over 0.8 across all age and ICU length of stay subgroups except for the subgroup with an ICU length of stay of more than 8 days in the AmsterdamUMCdb. Finally, all the validations in this study were conducted retrospectively. Therefore, unavoidable bias may occur, and prospective validation is required. Whether predicting mortality in ICU can improve outcomes should also be evaluated in future studies.

## Conclusions

In conclusion, we successfully developed a real-time ensemble machine learning model to predict short-term mortality in the ICU. This model was trained using a single-center dataset from South Korea. However, external validation using the publicly available MIMIC-III, eICU-CRD, and AmsterdamUMCdb showed that the model performance was reliably maintained across international cohorts. If our results are confirmed in future prospective studies, this model has the potential to serve as a useful decision-support tool when monitoring real-time risk in ICU patients.

### Supplementary Information

**Additional file 1**. Table E1-7 and Figure E1-10.

## Data Availability

The datasets generated during the current study are not publicly available but are available from the corresponding author on reasonable request.

## Abbreviations

ICU: Intensive care unit
GBM: Gradient boosting machine
LSTM: Long short-term memory
IRB: Institutional Review Board
SNUH: Seoul National University Hospital
MIMIC: Medical information mart for intensive care
eICU-CRD: EICU-Collaborative research database
AmsterdamUBCdb: Amsterdam University Medical Center database
MICU: Medical intensive care unit
SICU: Surgical intensive care unit
CCU: Coronary care unit
AUROC: Area under the receiver operating characteristic curve
DL: Deep learning
ECE: Expected calibration error
SPTTS: Single-parameter weighted track and trigger system
MEWS: Modified early warning score
NEWS: National early warning score
APACHE: Acute physiology and chronic health evaluation
SAPS: Simplified acute physiology score
SOFA: Sequential organ failure assessment
AUPRC: Area under the precision-recall curve
MACPD: Mean alarm count per day
SpO_2_: Saturation of peripheral oxygen
GCS: Glasgow coma scale

## References

[1] Shickel B, Loftus TJ, Adhikari L, Ozrazgat-Baslanti T, Bihorac A, Rashidi P (2019). DeepSOFA: a continuous acuity score for critically ill patients using clinically interpretable deep learning. Sci Rep.

[2] Iwase S, Nakada TA, Shimada T, Oami T, Shimazui T, Takahashi N (2022). Prediction algorithm for ICU mortality and length of stay using machine learning. Sci Rep.

[3] Delahanty RJ, Kaufman D, Jones SS (2018). Development and evaluation of an automated machine learning algorithm for in-hospital mortality risk adjustment among critical care patients. Crit Care Med.

[4] Baker S, Xiang W, Atkinson I (2020). Continuous and automatic mortality risk prediction using vital signs in the intensive care unit: a hybrid neural network approach. Sci Rep.

[5] Yoon HK, Yang HL, Jung CW, Lee HC (2022). Artificial intelligence in perioperative medicine: a narrative review. Korean J Anesthesiol.

[6] Thorsen-Meyer HC, Nielsen AB, Nielsen AP, Kaas-Hansen BS, Toft P, Schierbeck J (2020). Dynamic and explainable machine learning prediction of mortality in patients in the intensive care unit: a retrospective study of high-frequency data in electronic patient records. Lancet Digit Health.

[7] Churpek MM, Yuen TC, Huber MT, Park SY, Hall JB, Edelson DP (2012). Predicting cardiac arrest on the wards: a nested case-control study. Chest.

[8] Knaus WA, Draper EA, Wagner DP, Zimmerman JE (1985). APACHE II: a severity of disease classification system. Crit Care Med.

[9] Le Gall JR, Lemeshow S, Saulnier F (1993). A new Simplified Acute Physiology Score (SAPS II) based on a European/North American multicenter study. JAMA.

[10] Andersen SK, Montgomery CL, Bagshaw SM (2020). Early mortality in critical illness—A descriptive analysis of patients who died within 24 hours of ICU admission. J Crit Care.

[11] Kakkera KSS, Chada A, Chatterjee K, Colaco C (2016). Mortality in the ICU: Who dies within the first 24 hours?. Chest.

[12] Ferryman K, Mackintosh M, Ghassemi M (2023). Considering biased data as informative artifacts in AI-assisted health care. N Engl J Med.

[13] Keuning BE, Kaufmann T, Wiersema R, Granholm A, Pettila V, Moller MH (2020). Mortality prediction models in the adult critically ill: a scoping review. Acta Anaesthesiol Scand.

[14] Johnson AE, Pollard TJ, Shen L, Lehman LW, Feng M, Ghassemi M (2016). MIMIC-III, a freely accessible critical care database. Sci Data.

[15] Pollard TJ, Johnson AEW, Raffa JD, Celi LA, Mark RG, Badawi O (2018). The eICU collaborative research database, a freely available multi-center database for critical care research. Sci Data.

[16] Hochreiter S, Schmidhuber J (1997). Long short-term memory. Neural Comput.

[17] Somepalli G, Goldblum M, Schwarzschild A, Bruss CB, Goldstein T. Saint: Improved neural networks for tabular data via row attention and contrastive pre-training. arXiv preprint arXiv. 2021;2106.01342.

[18] Kwon Jm, Lee Y, Lee Y, Lee S, Park J. An algorithm based on deep learning for predicting in‐hospital cardiac arrest. JAHA. 2018;7:e008678. 10.1161/JAHA.118.008678 PMC6064911 29945914

[19] Akiba T, Sano S, Yanase T, Ohta T, Koyama M: Optuna: a next-generation hyperparameter optimization framework. In: Proceedings of the 25th ACM SIGKDD international conference on knowledge discovery & data mining: 2019;2019:2623–31.

[20] Srivastava N, Hinton G, Krizhevsky A, Sutskever I, Salakhutdinov R (2014). Dropout: a simple way to prevent neural networks from overfitting. J Mach Learn Res.

[21] Lee YJ, Cho K-J, Kwon O, Park H, Lee Y, Kwon J-M (2021). A multicentre validation study of the deep learning-based early warning score for predicting in-hospital cardiac arrest in patients admitted to general wards. Resuscitation.

[22] Kang Y, Jia X, Wang K, Hu Y, Guo J, Cong L (2020). A clinically practical and interpretable deep model for ICU mortality prediction with external validation. AMIA Annu Symp Proc.

[23] Meiring C, Dixit A, Harris S, MacCallum NS, Brealey DA, Watkinson PJ (2018). Optimal intensive care outcome prediction over time using machine learning. PLoS ONE.

[24] Meyer A, Zverinski D, Pfahringer B, Kempfert J, Kuehne T, Sundermann SH (2018). Machine learning for real-time prediction of complications in critical care: a retrospective study. Lancet Respir Med.

[25] Pirracchio R, Petersen ML, Carone M, Rigon MR, Chevret S, van der Laan MJ (2015). Mortality prediction in intensive care units with the Super ICU Learner Algorithm (SICULA): a population-based study. Lancet Respir Med.

[26] Cox EGM, Wiersema R, Eck RJ, Kaufmann T, Granholm A, Vaara ST (2023). External validation of mortality prediction models for critical illness reveals preserved discrimination but poor calibration. Crit Care Med.

[27] Schmid F, Goepfert MS, Reuter DA (2013). Patient monitoring alarms in the ICU and in the operating room. Crit Care.

[28] Covino M, Sandroni C, Della Polla D, De Matteis G, Piccioni A, De Vita A (2023). Predicting ICU admission and death in the Emergency Department: a comparison of six early warning scores. Resuscitation.

