# Supplementary material for: Real-time machine learning model to predict short-term mortality in critically ill patients: development and international validation
Source: Crit Care. 2024 Mar 14;28:76. doi: 10.1186/s13054-024-04866-7 (PMC10938661; doi:10.1186/s13054-024-04866-7)
Supplement: Supplementary file 1 — Additional file 1. Table E1-7 and Figure E1-10. [file 13054_2024_4866_MOESM1_ESM.docx]

**Real-time machine learning model to predict short-term mortality in critically ill patients: development and international validation**

Leerang Lim, MD^1*^, Ukdong Gim, MS^2*^, Kyungjae Cho, MS^2^, Dongjoon Yoo, MD^2,3^, Ho Geol Ryu, MD, PhD^1,4^, Hyung-Chul Lee, MD, PhD^1†^

^1^ Department of Anesthesiology and Pain Medicine, Seoul National University College of Medicine, Seoul National University Hospital, 101 Daehak-ro, Jongno-gu, Seoul, 03080, Republic of Korea

^2^ VUNO, 479 Gangnam-daero, Seocho-gu, Seoul, 06541, Republic of Korea

^3^ Department of Critical Care Medicine and Emergency Medicine, Inha University College of Medicine, 100 Inha-ro, Michuhol-gu, Incheon, 22212, Republic of Korea

^4^ Department of Critical Care Medicine, Seoul National University College of Medicine, Seoul National University Hospital, 101 Daehak-ro, Jongno-gu, Seoul, 03080, Republic of Korea

**Table E1**. 30 candidate and 16 selected laboratory variables for model development

| Candidate variables | Selected variables |
| --- | --- |
| Activated partial thromboplastin time | Activated partial thromboplastin time |
| Alanine aminotransferase | Alanine aminotransferase |
| Albumin | Albumin |
| Alkaline phosphate |  |
| Aspartate aminotransferase | Aspartate aminotransferase |
| Bilirubin | Bilirubin |
| Blood urea nitrogen | Blood urea nitrogen |
| Brain natriuretic peptide |  |
| Calcium |  |
| Chloride | Chloride |
| C-reactive protein | C-reactive protein |
| Creatinine | Creatinine |
| Creatinine kinase |  |
| Creatinine kinase muscle brain | Sodium |
| Erythrocyte Sedimentation Rate |  |
| Glucose | Glucose |
| Hemoglobin | Hemoglobin |
| Partial pressure of carbon dioxide pressure |  |
| Partial pressure of oxygen |  |
| Platelets | Platelets |
| Potassium | Potassium |
| Protein |  |
| Prothrombin time (INR) | Prothrombin time (INR) |
| Prothrombin time (sec) |  |
| Red blood cell |  |
| Sodium |  |
| Total cholesterol |  |
| Troponin I |  |
| Urinalysis |  |
| White blood cell | White blood cell |

**Table E2.** Baseline characteristics of the study cohorts

|  | **Survival group** | | | | **Mortality group** | | | |
| --- | --- | --- | --- | --- | --- | --- | --- | --- |
|  | **SNUH** | **MIMIC-III** | **eICU-CRD** | **AmsterdamUMCdb** | **SNUH** | **MIMIC** | **eICU-CRD** | **AmsterdamUMCdb** |
| Number of admissions (%) | 80066 (99%) | 42501 (97%) | 165421 (98%) | 13279 (97%) | 1120 (1%) | 1503 (3%) | 3663 (2%) | 354 (3%) |
| Number of samples (%) | 9026225 (99%) | 5943127 (99%) | 15847502 (99%) | 904443 (98%) | 113799 (1%) | 62095 (1%) | 149646 (1%) | 16329 (2%) |
| Gender (male/female) | 49033/31033 | 24607/17894 | 90819/74602 | 8837/4442 | 679/441 | 841/662 | 2115/1548 | 233/121 |
| Age (year) | 61.8 ± 14.6 | 62.1 ± 17.0 | 61.5 ± 16.9 | 59.4 ± 15.57 | 61.6 ± 15.7 | 67.1 ± 16.3 | 65.6 ± 16.2 | 63.34 ± 13.92 |
| *9 vital signs* |  |  |  |  |  |  |  |  |
| Respiratory rate (/min) | 19.7 ± 6.4 | 19.9 ± 6.0 | 19.7 ± 5.9 | 18.09 ± 6.41 | 22.6 ± 7.8 | 22.2 ± 6.9 | 21.9 ± 6.71 | 22.58 ± 6.94 |
| Heart rate (/min) | 86.4 ± 19.8 | 86.1 ± 17.6 | 85.8 ± 18.4 | 81.82 ± 17.07 | 96.5 ± 24.5 | 91.5 ± 19.3 | 93.3 ± 21.1 | 92.15 ± 22.77 |
| SBP (mmHg) | 125.6 ± 24.7 | 123.5 ± 23.2 | 123.6 ± 23.5 | 126.89 ± 25.06 | 114.1 ± 27.5 | 113.3 ± 23.9 | 113.1 ± 25.1 | 116.64 ± 30.85 |
| DBP (mmHg) | 70.6 ± 13.7 | 61.6 ± 14.35 | 6­­­4.9 ± 14.9 | 62.91 ± 12.83 | 66.0 ± 16.1 | 57.3 ± 13.8 | 59.2 ± 15.0 | 60.64 ± 14.74 |
| Body temperature (℃) | 36.8 ± 0.7 | 37.1 ± 0.8 | 37.0 ± 0.7 | 36.71 ± 0.86 | 36.4 ± 1.1 | 36.9 ± 1.0 | 36.7 ± 1.3 | 36.09 ± 1.63 |
| SpO_2_ (%) | 98.2 ± 2.5 | 97.24 ± 2.7 | 96.8 ± 2.9 | 96.93 ± 2.88 | 95.9 ± 5.8 | 97.0 ± 3.9 | 96.2 ± 4.37 | 95.88 ± 4.21 |
| GCS– eye | 3.2 ± 1.1 | 3.51 ± 0.9 | 3.6 ± 0.8 | 3.41 ± 0.93 | 2.3 ± 1.3 | 2.8 ± 1.2 | 2.6 ± 1.3 | 1.65 ± 1.14 |
| GCS – verbal | 4.5 ± 1.0 | 4.58 ± 1.0 | 3.8 ± 1.7 | 4.48 ± 1.14 | 3.8 ± 1.4 | 3.7 ± 1.6 | 2.1 ± 1.6 | 2.17 ± 1.59 |
| GCS – motor | 5.2 ± 1.6 | 5.46 ± 1.2 | 5.7 ± 1.0 | 5.51 ± 1.23 | 3.6 ± 2.2 | 4.0 ± 2.0 | 4.0 ± 2.1 | 3.06 ± 2.22 |
| *16 laboratory results* |  |  |  |  |  |  |  |  |
| ALT (Units/L) | 83.7 ± 220.0 | 187.0 ± 505.2 | 118.8 ± 377.6 | 99.05 ± 279.46 | 206.1 ± 488.4 | 313.5 ± 703.9 | 322.51 ± 718.2 | 239.26 ± 432.86 |
| AST (Units/L) | 90.5 ± 270.9 | 236.6 ± 759.6 | 159.0 ± 583.6 | 136.07 ± 435.77 | 271.9 ± 720.8 | 558.8 ± 1371.5 | 506.9 ± 1234.2 | 329.59 ± 439.05 |
| Albumin (g/dL) | 3.0 ± 0.5 | 2.9 ± 0.7 | 2.7 ± 0.7 | 2.4 ± 0.57 | 2.8 ± 0.5 | 2.7 ± 0.7 | 2.5 ± 0.7 | 2.21 ± 0.64 |
| BUN (mg/dL) | 25.3 ± 18.8 | 28.8 ± 22.6 | 27.2 ± 21.3 | 24.07 ± 18.28 | 36.2 ± 23.6 | 42.0 ± 27.2 | 39.6 ± 26.2 | 31.41 ± 21.99 |
| Bilirubin (mg/dL) | 2.0 ± 3.3 | 3.0 ± 5.3 | 1.5 ± 3.1 | 0.84 ± 1.36 | 6.1 ± 8.4 | 7.1 ± 9.9 | 3.6 ± 6.4 | 1.8 ± 3.36 |
| CRP (mg/dL) | 7.9 ± 7.6 | 9.9 ± 8.4 | 20.1 ± 40.0 | 8.54 ± 9.22 | 11.7 ± 8.5 | 12.4 ± 7.7 | 21.1 ± 44.5 | 10.97 ± 9.22 |
| Chloride (mmol/L) | 104.8 ± 6.4 | 104.9 ± 6.2 | 105.0 ± 7.2 | 107.77 ± 5.06 | 103.4 ± 8.2 | 104.5 ± 7.8 | 105.7 ± 7.9 | 106.5 ± 6.28 |
| Creatinine (mg/dL) | 1.3 ± 1.36 | 1.46 ± 1.49 | 1.48 ± 1.5 | 1.17 ± 1.16 | 1.59 ± 1.17 | 2.0 ± 1.6 | 2.0 ± 1.5 | 1.79 ± 1.15 |
| Glucose (mg/dL) | 161.6 ± 62.2 | 133.0 ± 46.8 | 147.4 ± 58.8 | 146.42 ± 42.21 | 164.1± 74.0 | 142.8 ± 64.5 | 152.6 ± 65.1 | 159.66 ± 71.51 |
| Hemoglobin (g/dL) | 10.5 ± 1.9 | 10.2 ± 1.7 | 10.3 ± 2.2 | 10.87 ± 1.76 | 9.7 ± 1.9 | 9.9 ± 1.8 | 10.1 ± 2.3 | 10.51 ± 1.92 |
| Prothrombin time (INR) | 1.3 ± 0.4 | 1.5 ± 0.7 | 1.6 ± 0.8 | 1.39 ± 0.4 | 1.7 ± 0.8 | 1.9 ± 1.0 | 1.9 ± 1.1 | 1.89 ± 0.96 |
| Platelets (10^3^/µL) | 167.1 ± 103.3 | 224.1 ± 138.0 | 203.5 ± 110.0 | 194.83 ± 111.73 | 105.7 ± 83.6 | 163.6 ± 131.8 | 150.3 ± 101.6 | 145.58 ± 129.52 |
| Potassium (mmol/L) | 4.0 ± 0.6 | 4.1 ± 0.62 | 4.0 ± 0.6 | 4.15 ± 0.47 | 4.1 ± 0.8 | 4.3 ± 0.8 | 4.3 ± 0.9 | 4.39 ± 0.82 |
| Sodium (mmol/L) | 138.0 ± 5.7 | 138.8 ± 5.1 | 139.0 ± 5.9 | 139.33 ± 4.43 | 139.0 ± 8.0 | 138.8 ± 6.7 | 140.8 ± 7.3 | 140.77 ± 6.19 |
| WBC (10^3^/µL) | 11.2 ± 5.9 | 11.7 ± 6.3 | 11.8 ± 6.4 | 13.26 ± 5.45 | 11.8 ± 8.6 | 13.83 ± 8.7 | 15.2 ± 10.2 | 14.49 ± 8.94 |
| aPTT (sec) | 39.4 ± 18.1 | 42.5 ± 23.9 | 45.6 ± 26.3 | 44.04 ± 18.43 | 52.74 ± 24.47 | 52.46 ± 29.5 | 51.9 ± 28.4 | 78.06 ± 62.36 |

SNUH=Seoul National University Hospital, MIMIC=Mart for Intensive Care, eICU-CRD=eICU Collaborative Research Database, AmsterdamUMCdb=Amsterdam University Medical Center database, SBP=systolic blood pressure, DBP=diastolic blood pressure, SpO_2_=Saturation of peripheral oxygen, GCS=Glasgow coma scale, ALT=Alanine aminotransferase, AST=Aspartate aminotransferase, BUN=Blood urea nitrogen, CRP=C-reactive protein, INR=International normalized ratio, WBC=White blood cell, aPTT=Activated partial thromboplastin time.

The data is represented by the number of samples (%) or the mean and ± standard deviation. The percentage adds up to 100 for both the survival and mortality groups within each cohort. All p-values were < 0.001 except for the activated partial thromboplastin time in the mortality group (p=0.062).

**Table E3.** Valid ranges and missing rates of input features

|  | Valid ranges | Internal development dataset missing rate (%) |
| --- | --- | --- |
| 9 vital signs |  |  |
| Respiratory rate (/min) | 5~50 | 28.47​ |
| Heart rate (/min) | 10~190​ | 22.55​ |
| Systolic blood pressure (mmHg) | 40~230​ | 31.84​ |
| Diastolic blood pressure (mmHg) | 20~130​ | 31.86​ |
| Body temperature (℃) | 32~41​ | 52.07​ |
| Saturation of peripheral oxygen (%) | 68~100​ | 26.28​ |
| Glasgow coma scale - eye | 1~4​ | 27.97​ |
| Glasgow coma scale - verbal | 1~5​ | 64.39​ |
| Glasgow coma scale - motor | 1~6​ | 27.96​ |
| 16 laboratory results | ​​ | ​ |
| Alanine aminotransferase (Units/L) | 0~5000​ | 97.11​ |
| Aspartate aminotransferase (Units/L) | 0~10000​ | 97.11​ |
| Albumin (g/dL) | 0~5.5​ | 97.09​ |
| Blood urea nitrogen (mg/dL) | 0~150​ | 96.34​ |
| Bilirubin (mg/dL) | 0~50​ | 97.16​ |
| C-reactive protein (mg/dL) | 0~300​ | 98.00​ |
| Chloride (mmol/L) | 70~135​ | 95.64​ |
| Creatinine (mg/dL) | 0~15​ | 96.31​ |
| Glucose (mg/dL) | 0~600​ | 92.16​ |
| Hemoglobin (g/dL) | 0~25​ | 96.38​ |
| Prothrombin time (INR) | 0~8​ | 97.56​ |
| Platelets (10^3^/µL) | 0~1000​ | 96.48​ |
| Potassium (mmol/L) | 2~9​ | 93.25​ |
| Sodium (mmol/L) | 105~170​ | 93.25​ |
| White blood cell (10^3^/µL) | 0~90​ | 96.37​ |
| Activated partial thromboplastin time (sec) | 0~300​ | 97.61​ |

INR=International normalized ratio.

**Table E4. Hyperparameters of the LSTM-based deep learning model for use in iMORS.**

| Parameter | Search space | Selected value |
| --- | --- | --- |
| Hidden dimension | [16, 32, 64, 128, 256] | 32 |
| Hidden dimension  (Feature-wise embedding layers) | - | 16 |
| Under-sampling ratio (non-event) | [1, 2, 4] | 2 |
| Dropout (LSTM) | [0.4, 0.5, 0.6, 0.7] | 0.4 |
| Dropout (Fully connected layers) | [0.4, 0.5, 0.6] | 0.5 |
| Optimizer | [Adam, Adamw] | Adamw |
| Batch size | [32, 64, 128, 256] | 128 |
| Number of fully connected layers | [0, 1, 2, 3, 4, 5] | 5 |
| Number of convolutional neural network layers | [0, 1, 2, 3, 4] | 0 |
| 3 LSTM layers | [Use, Not use] | Use |

LSTM=long short-term memory, Adam=Adaptive moment estimation, Adamw=Adaptive moment estimation with weight decay.

| Table E5. Categorized ICU type by specialty of Amsterdam University Medical Center database | | |
| --- | --- | --- |
| **Specialty (original)** | **Specialty (transrated)** | **ICU type** |
| Cardiochirurgie | Cardiothoracic Surgery | SICU |
| Neurochirurgie | Neurosurgery | SICU |
| Vaatchirurgie | Vascular Surgery | SICU |
| Traumatologie | Traumatology | SICU |
| Inwendig | Internal Medicine | MICU |
| Heelkunde Gastro-enterologie | Surgery Gastroenterology | SICU |
| Cardiologie | Cardiology | CCU |
| Neurologie | Neurology | MICU |
| Intensive Care Volwassenen | Adult Intensive Care | MICU |
| Heelkunde Oncologie | Surgery Oncology | SICU |
| Nefrologie | Nephrology | MICU |
| Heelkunde Longen/Oncologie | Surgery Lungs/Oncology | SICU |
| Longziekte | Pulmonology | MICU |
| Keel, Neus & Oorarts | Otorhinolaryngology (ENT) | SICU |
| Urologie | Urology | SICU |
| Orthopedie | Orthopedics | SICU |
| Hematologie | Hematology | MICU |
| Maag-,Darm-,Leverziekten | Gastroenterology | MICU |
| Gynaecologie | Gynecology | SICU |
| Oncologie Inwendig | Internal Oncology | MICU |
| Plastische chirurgie | Plastic Surgery | SICU |
| Mondheelkunde | Oral and Maxillofacial Surgery | SICU |
| Obstetrie | Obstetrics | SICU |
| Verloskunde | Midwifery | SICU |
| Reumatologie | Rheumatology | MICU |
| Oogheelkunde | Ophthalmology | SICU |

MICU=medical intensive care unit, SICU=surgical intensive care unit, CCU=coronary care unit.

**Table E6.** The area under the receiver operating characteristic (AUROC) curves for the cohorts

|  | SNUH | | MIMIC-III | | eICU-CRD | | AmsterdamUMCdb | |
| --- | --- | --- | --- | --- | --- | --- | --- | --- |
|  | **AUROC (95% CI)** | **p-value** | **AUROC (95% CI)** | **p-value** | **AUROC (95% CI)** | **p-value** | **AUROC** **(95% CI)** | **p-value** |
| **iMORS** | 0.964 (0.963-0.965)​ | - | 0.890 (0.889-0.891)​ | - | 0.886  (0.885-0.887)​ | - | 0.870  (0.868-0.873)​ | - |
| **MEWS**​ | 0.781  (0.777-0.785)​ | <0.001​ | 0.731  (0.729-0.733)​ | <0.001​ | 0.801  (0.800-0.802)​ | <0.001​ | 0.821  (0.819-0.824)​ | <0.001​ |
| **NEWS**​ | 0.866  (0.863-0.869)​ | <0.001 | 0.746  (0.745-0.748)​ | <0.001 | 0.798  (0.797-0.799)​ | <0.001 | 0.819  (0.817-0.822)​ | <0.001 |
| **APACHE-II**​ | 0.831  (0.829-0.834)​ | <0.001​ | 0.776  (0.774-0.777)​ | <0.001​ | 0.815  (0.814-0.816)​ | <0.001​ | 0.805  (0.802-0.809)​ | <0.001​ |
| **SOFA**​ | 0.785  (0.782-0.788)​ | <0.001​ | 0.712  (0.711-0.714)​ | <0.001​ | 0.769  (0.768-0.770)​ | <0.001​ | 0.784  (0.781-0.788)​ | <0.001​ |
| **SAPS-II**​ | 0.840  (0.837-0.844)​ | <0.001​ | 0.758  (0.756-0.759)​ | <0.001​ | 0.815  (0.813-0.816)​ | <0.001​ | 0.824  (0.821-0.828)​ | <0.001​ |

SNUH=Seoul National University Hospital, MIMIC-III=Mart for Intensive Care-III, eICU-CRD=eICU Collaborative Research Database, AmsterdamUMCdb=Amsterdam University Medical Center database, SPTTS=single-parameter weighted “track and trigger” systems, NEWS=national early warning score, MEWS=modified early warning score, APACHE=acute physiology and chronic health evaluation, SAPS=simplified acute physiology score, SOFA=sequential organ failure assessment, AUROC=area under the receiver operating characteristic, CI=confidence interval.

The *p*-value of the AUROC score between iMORS and other models was calculated using the Delong test. iMORS denotes our model.

**Table E7.** The mean alarm count per day (MACPD) at the same sensitivity for the study cohorts.

| **SNUH** | | | | | **MIMIC-III** | | | | | **eICU-CRD** | | | | | **AmsterdamUMCdb** | | | | |
| --- | --- | --- | --- | --- | --- | --- | --- | --- | --- | --- | --- | --- | --- | --- | --- | --- | --- | --- | --- |
| **Cutoff** | **Sens** | **MACPD** | **Alarm rate** | **p-value** | **Cutoff** | **Sens** | **MACPD** | **Alarm rate** | **p-value** | **Cutoff** | **Sens** | **MACPD** | **Alarm rate** | **p-value** | **Cutoff** | **Sens** | **MACPD** | **Alarm rate** | **p-value** |
| **NEWS ≥ 4**​ | 0.891​ | 8.954​ | 100%​ | <0.001 | **NEWS ≥ 4**​ | 0.788​ | 10.645​ | 100%​ | <0.001 | **NEWS ≥ 4**​ | 0.888​ | 10.989​ | 100%​ | <0.001 | **NEWS ≥ 4​** | 0.783 | 10.142 | 100%​ | <0.001 |
| **iMORS≥ 37.8**​ |  | 2.213​ | 24.72%​ |  | **iMORS**​**≥ 27.8**​ |  | 5.193​ | 48.78%​ |  | **iMORS**​**≥ 22.3** |  | 9.174 | 83.48%​ |  | **iMORS​≥​ 31.4** |  | 6.509 | 64.18%​ |  |
| **NEWS ≥ 5**​ | 0.832​ | 5.736​ | 100%​ | <0.001 | **NEWS ≥ 5**​ | 0.696​ | 6.982​ | 100%​ | <0.001 | **NEWS ≥ 5**​ | 0.838​ | 8.123​ | 100%​ | <0.001 | **NEWS ≥ 5​** | 0.682 | 5.967 | 100%​ | <0.001 |
| **iMOR ≥ 45.1**​ |  | 1.537​ | 26.80%​ |  | **iMORS**​**≥ 34.4**​ |  | 2.901​ | 41.54%​ |  | **iMORS≥ 26.0** |  | 6.274​ | 77.25%​ |  | **iMORS​≥ 25.9​** |  | 3.685 | 61.75%​ |  |
| **NEWS ≥ 6**​ | 0.736​ | 3.366​ | 100%​ | <0.001 | **NEWS ≥ 6**​ | 0.590​ | 4.265​ | 100%​ | <0.001 | **NEWS ≥ 6**​ | 0.749​ | 5.416​ | 100%​ | <0.001 | **NEWS ≥ 6​** | 0.592 | 3.589 | 100%​ | <0.001 |
| **iMOR ≥ 56.5** |  | 0.955​ | 28.38%​ |  | **iMORS**​**≥ 42.1**​ |  | 1.438​ | 33.72%​ |  | **iMORS≥ 32.0**​ |  | 3.482​ | 64.29%​ |  | **iMORS≥​ 39.9** |  | 2.289 | 63.77%​ |  |
| **SPTTS**​ | 0.731​ | 7.494​ | 100%​ | <0.001 | **SPTTS**​ | 0.567​ | 5.965​ | 100%​ | <0.001 | **SPTTS**​ | 0.580​ | 5.266​ | 100%​ | <0.001 | **SPTTS​** | 0.589 | 7.769 | 100%​ | <0.001 |
| **iMORS≥ 57.1**​ |  | 0.937​ | 12.51%​ |  | **iMORS**​**≥ 43.8**​ |  | 1.239​ | 20.77%​ |  | **iMORS≥ 42.8**​ |  | 1.276​ | 24.24%​ |  | **iMORS≥ 40.0** |  | 2.259 | 29.08%​ |  |

NEWS=national early warning score, MEWS=modified early warning score, SNUH=Seoul National University Hospital, MIMIC-III=Mart for Intensive Care-III, eICU-CRD =eICU Collaborative Research Database, AmsterdamUMCdb=Amsterdam University Medical Center database, SPTTS=single-parameter weighted “track and trigger” systems, Sens=Sensitivity.

MACPD is determined to indicate the average alarm count per bed every day. The two-sample t-test was used for calculating the *p*-value between MACPD of comparison models and iMORS at specific cutoffs. iMORS denotes our model.

**Fig. E1.** The flow chart of the study.


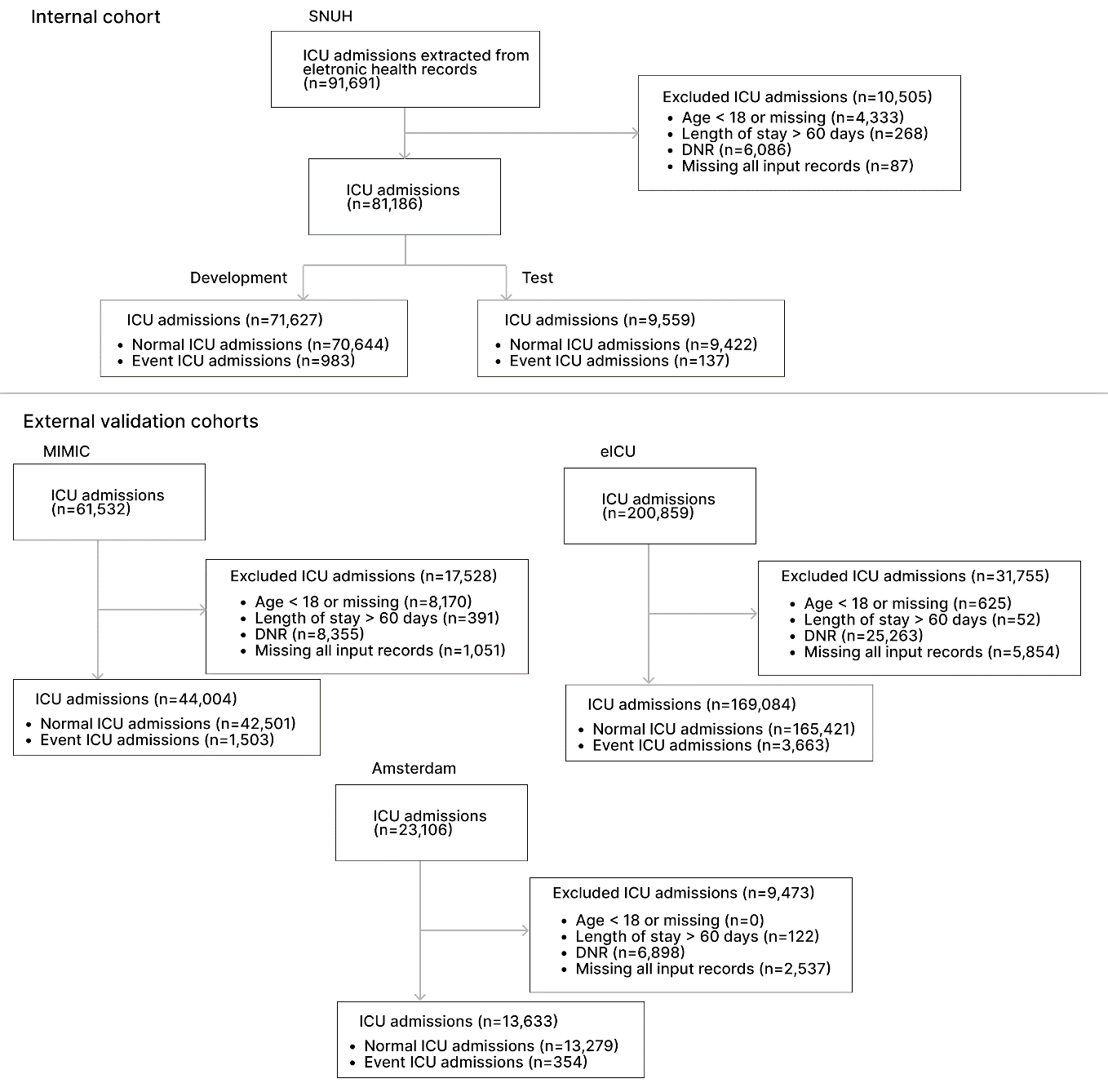

SNUH=Seoul National University Hospital, MIMIC=Mart for Intensive Care, eICU=eICU Collaborative Research Database, Amsterdam=Amsterdam University Medical Center database, ICU=intensive care unit, DNR=do not resuscitate,

**Fig. E2.** An overarching depiction of the study’s development and validation process.


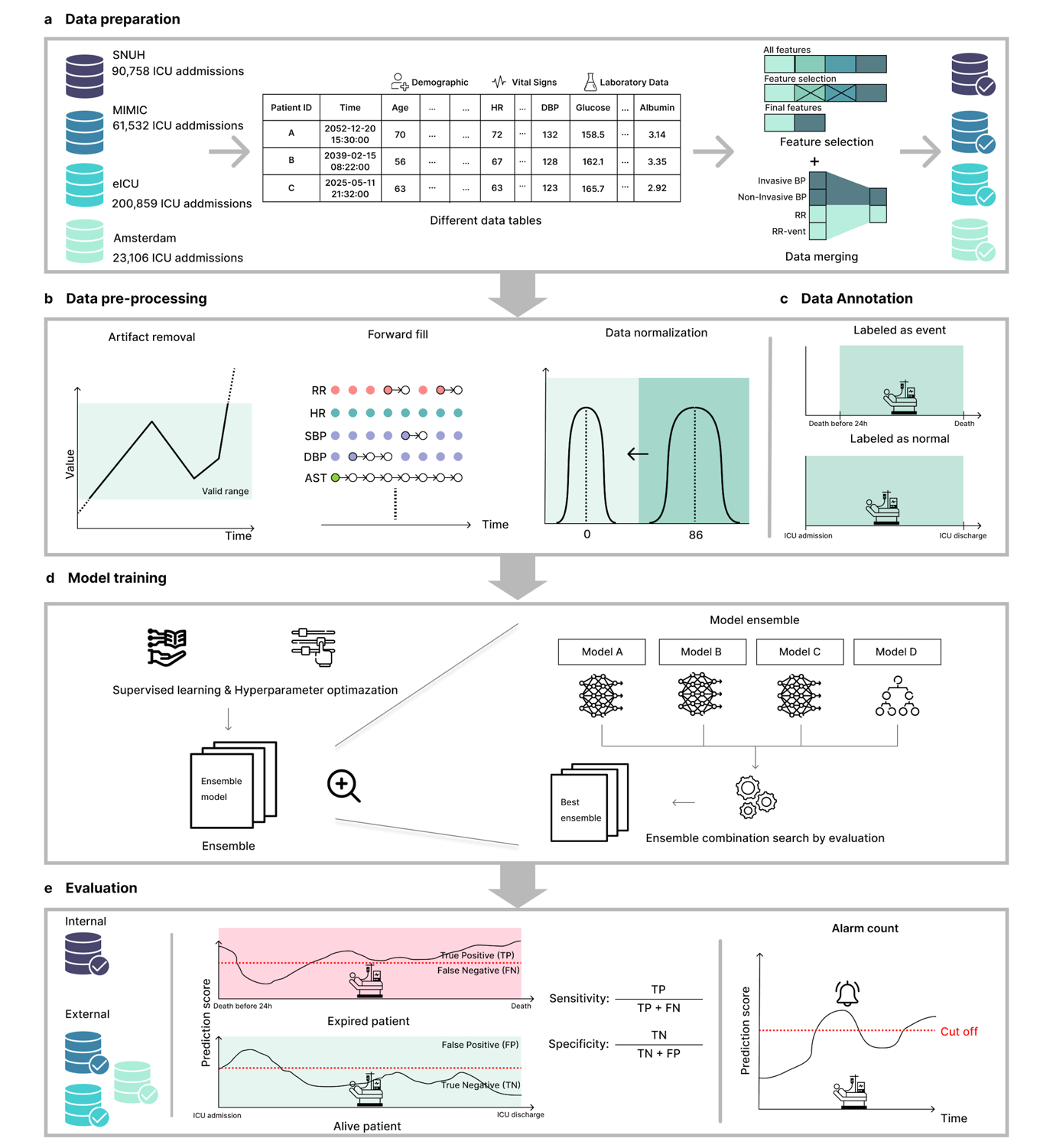


**a** Each cohort comprises multiple data tables, which are consolidated into a unified cohort. **b** Outlying values were treated as missing, and the initial missing value was substituted with the median value from the training set. Subsequent missing values were forward-filled. Each value underwent normalization using the mean and standard deviation derived from the training set. **c** Samples were obtained 24 hours before the event for the event admissions, whereas samples from the non-event admissions were classified as normal. **d** We trained multiple models, optimizing hyperparameters for each model, and assessed various model combinations to identify the optimal ensemble with the highest performance. e Model evaluation involved computing the area under the receiver operating characteristic curve for prediction scores and alarm counts. **f** Data distribution and challenging examples within each cohort were examined to enhance the exclusion criteria, tailoring them more effectively to the cohorts’ specific characteristics.

SNUH=Seoul National University Hospital, MIMIC=Mart for Intensive Care, eICU=eICU Collaborative Research Database, Amsterdam=Amsterdam University Medical Center database, HR=heart rate, RR=respiratory rate, SBP=systolic blood pressure, DBP=diastolic blood pressure, BP=blood pressure, AST=Aspartate aminotransferase, ICU=intensive care unit.

**Fig. E3.** The month-wise mortality in the intensive care unit of the internal cohort.


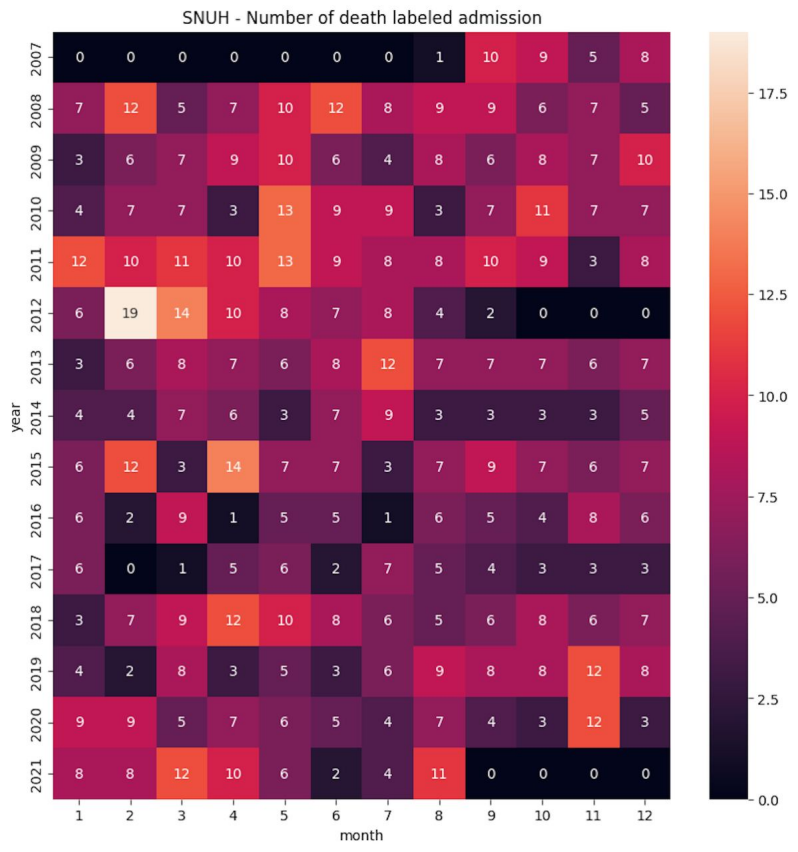


SNUH=Seoul National University Hospital.

**Fig. E4.** The distribution of National Early Warning Score (NEWS) in the cohorts.


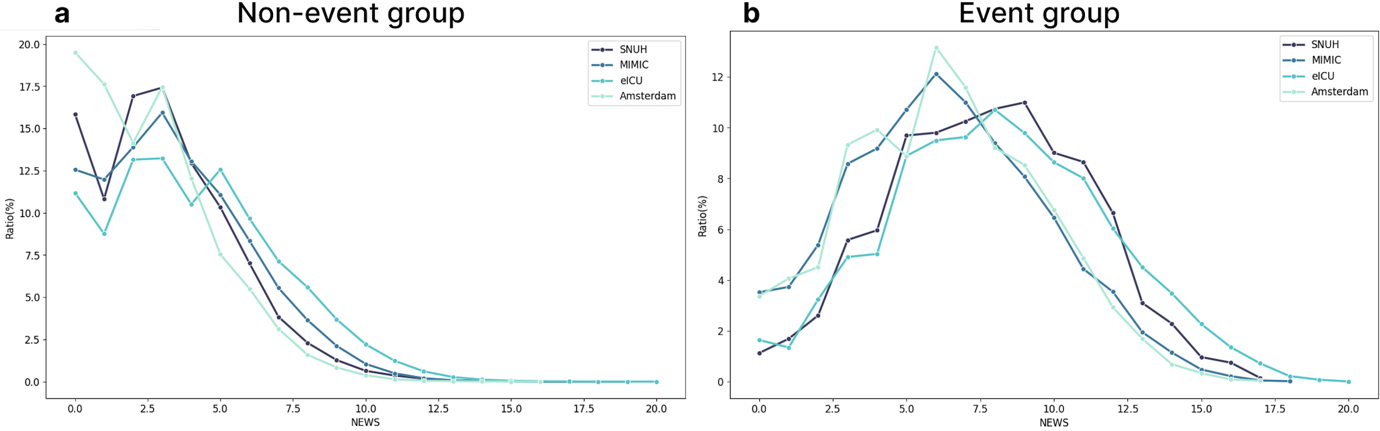
**a** The distribution of NEWS in non-event group. **b** The distribution of NEWS in event group.

The NEWS values, ranging from 1 to 20, are represented as integers. The y-axis indicates the percentage of specific scores within the normal or event data in the cohort.

NEWS=national early warning score, SNUH=Seoul National University Hospital, MIMIC=Mart for Intensive Care, eICU=eICU Collaborative Research Database, Amsterdam=Amsterdam University Medical Center database.

**Fig. E5.** The data distribution of 29 features in the cohorts.


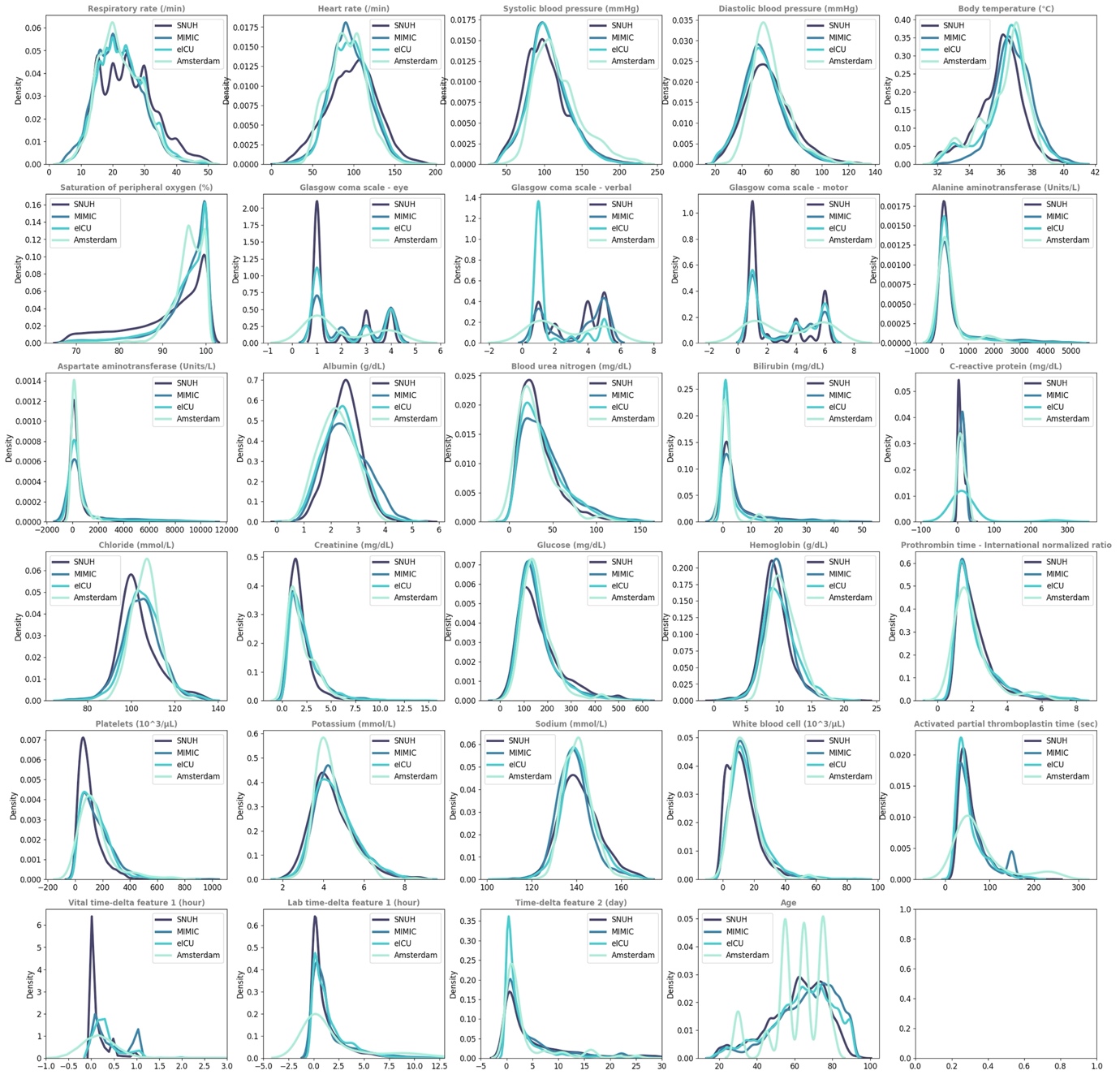
SNUH=Seoul National University Hospital, MIMIC=Mart for Intensive Care, eICU=eICU Collaborative Research Database, Amsterdam=Amsterdam University Medical Center database.

Outlier values have been excluded from the analysis. The features “Glasgow coma scale - eye, verbal, and motor” are represented as integers, while all other features are numeric values.

**Fig. E6.** The area under the precision-recall curves (AUPRCs) in the study cohorts.


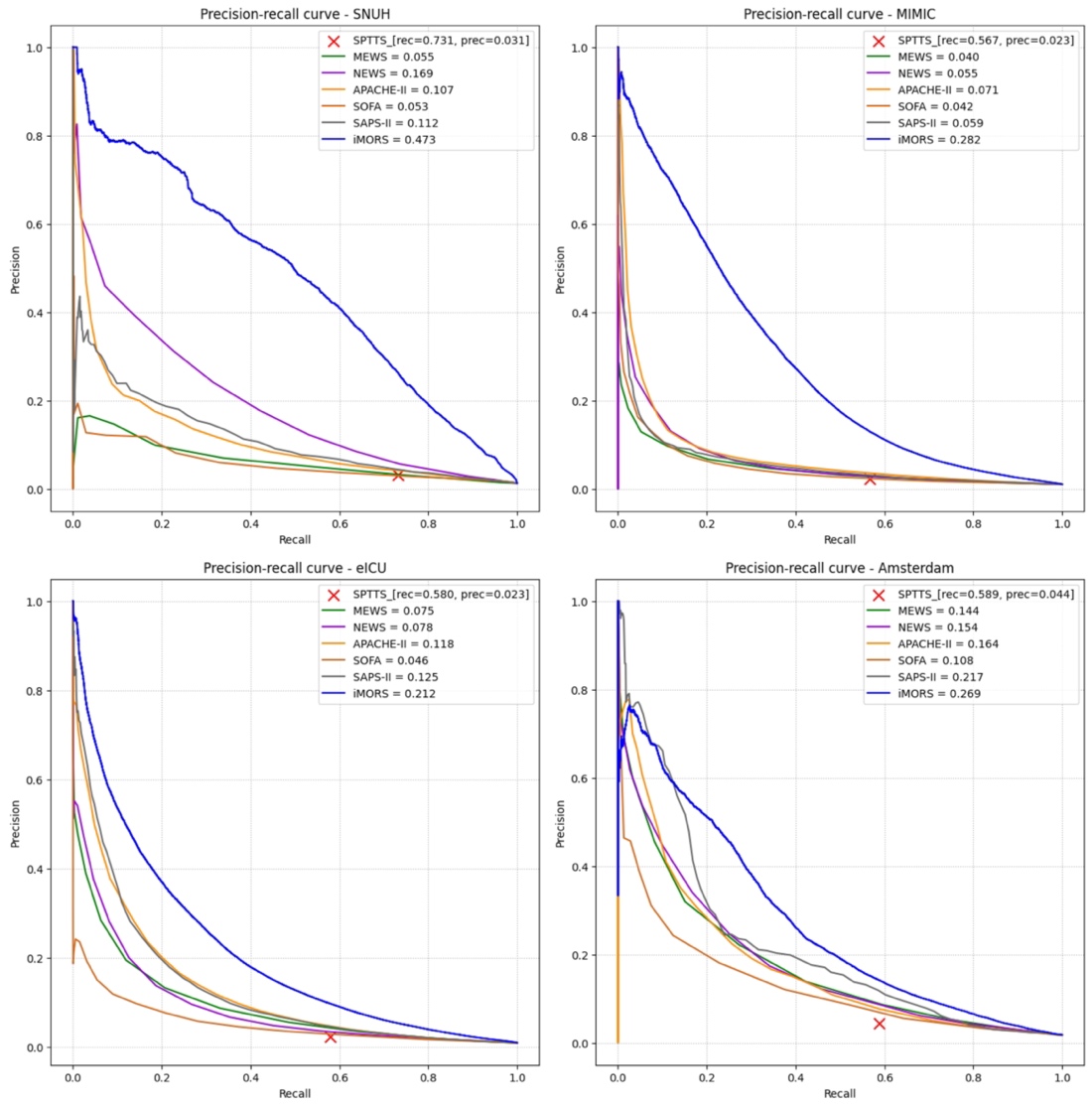


SNUH=Seoul National University Hospital, MIMIC=Mart for Intensive Care, eICU=eICU Collaborative Research Database, Amsterdam=Amsterdam University Medical Center database, SPTTS=single-parameter weighted “track and trigger” systems, NEWS=national early warning score, MEWS=modified early warning score, APACHE=acute physiology and chronic health evaluation, SAPS=simplified acute physiology score, SOFA=sequential organ failure assessment.

The designation “iMORS” represents our model, while the other listed models serve as comparative benchmarks.

**Fig. E7.** The Shapley summary plots.


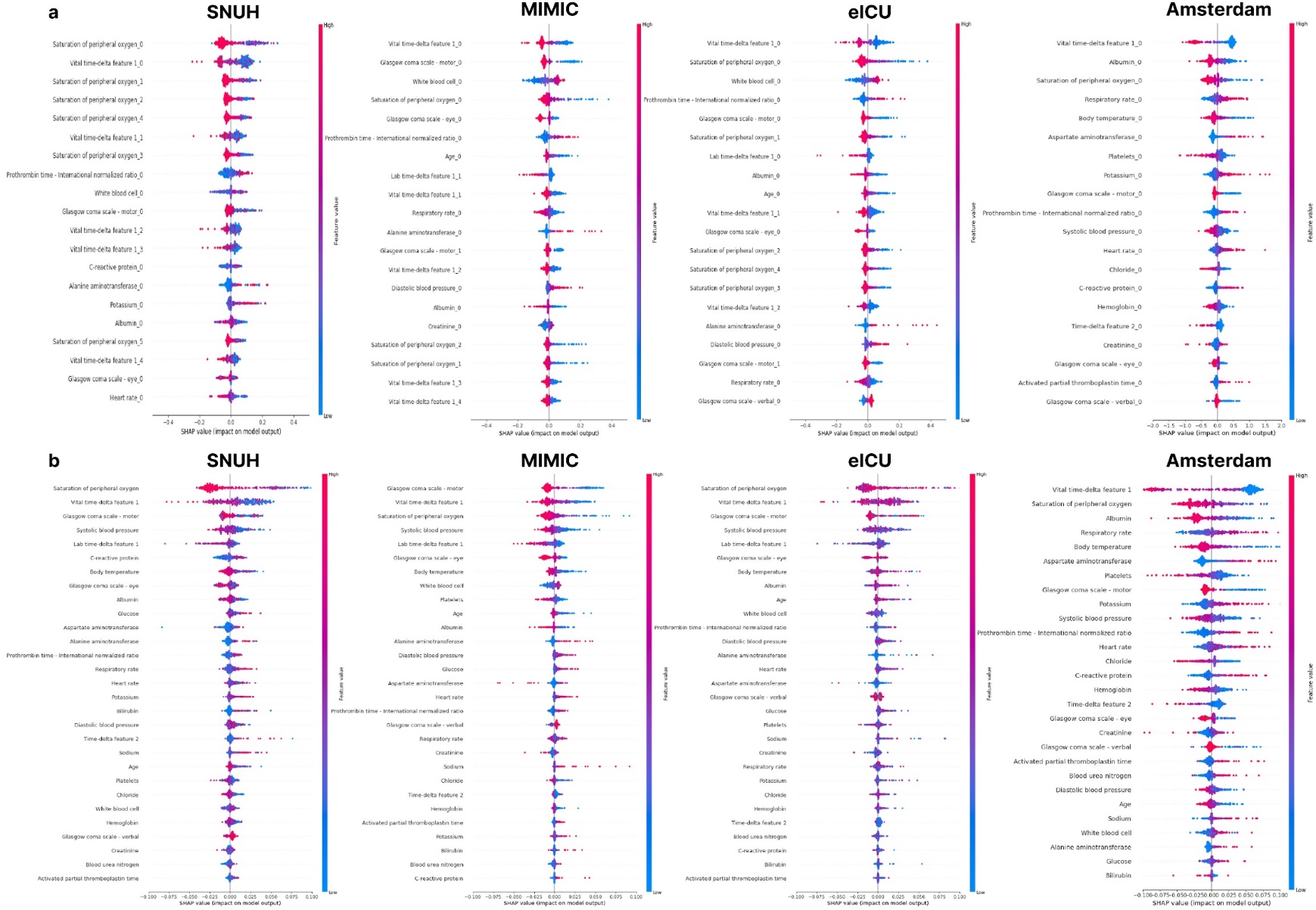


**a** The Shapley value for all features employed in the study, with the Shapley values and feature values averaged for each sequence data. **b** The top 20 Shapley values at each time step. The number following the ‘_’ symbol indicates the order of the sequence data, ranging from the current (0) to the most distant sample data. *Vital time-delta feature represents the elapsed time since the last measurement of any vital signs., Lab time-delta feature represents the elapsed time since the last measurement of any laboratory results, and ICU time-delta represents the elapsed time from the patient’s admission to the ICU.

ICU=intensive care unit, SNUH=Seoul National University Hospital, MIMIC=Mart for Intensive Care, eICU=eICU Collaborative Research Database, Amsterdam=Amsterdam University Medical Center database.

**Fig. E8.** Calibration curve for the total cohorts.


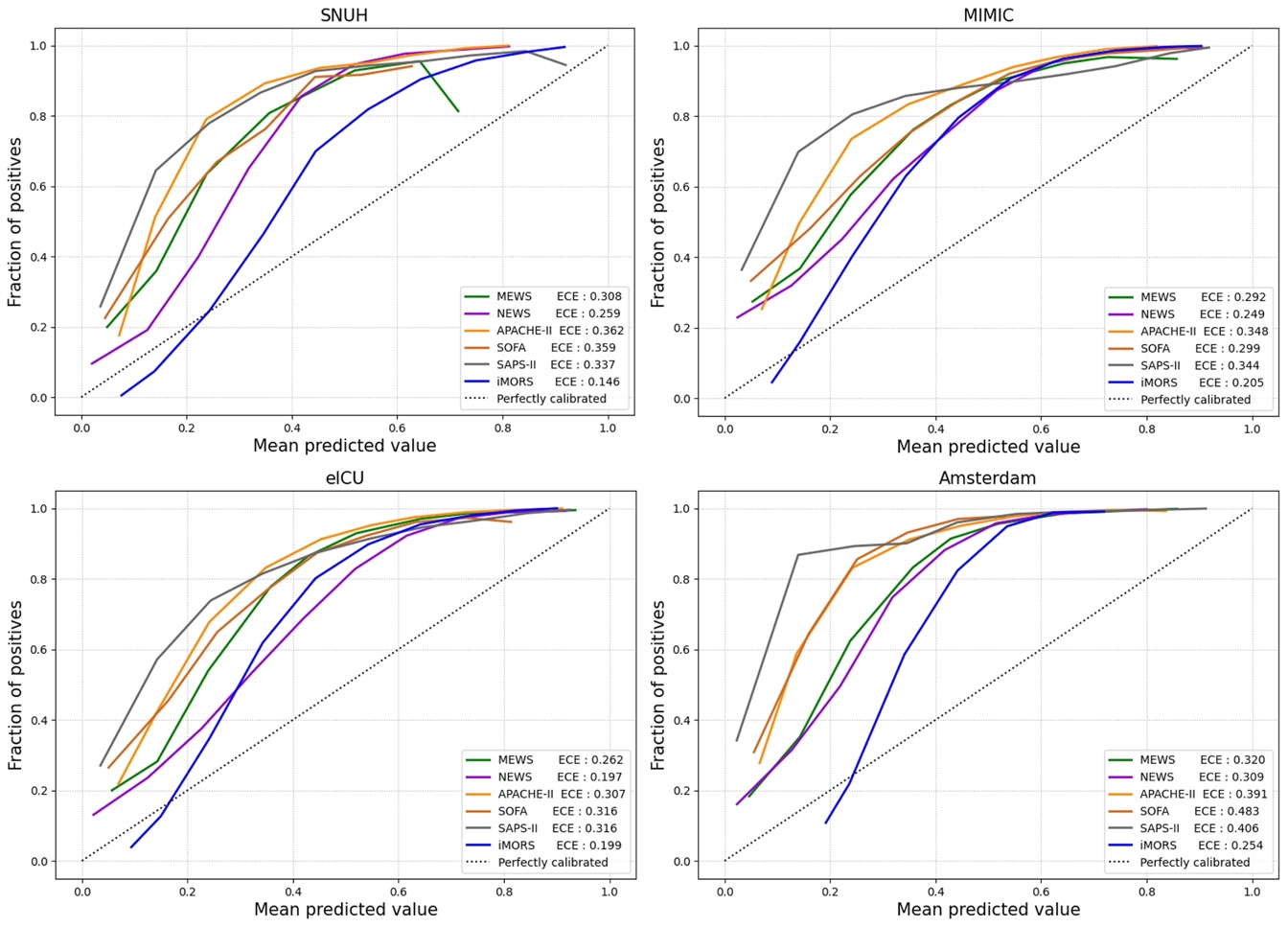


The Expected Calibration Error (ECE) is calculated as the average absolute difference between the mean predicted value and the positive sample fraction. The ratio of positive samples is taken into account when computing the mean predicted values. iMORS denotes our model.
SNUH=Seoul National University Hospital, MIMIC=Mart for Intensive Care, eICU=eICU Collaborative Research Database, Amsterdam=Amsterdam University Medical Center database, MEWS=modified early warning score, NEWS=national early warning score, APACHE=acute physiology and chronic health evaluation, SAPS=simplified acute physiology score, SOFA=sequential organ failure assessment.

**Fig. E9.** Subgroup analysis on external cohorts, evaluated using AUROC measurements.


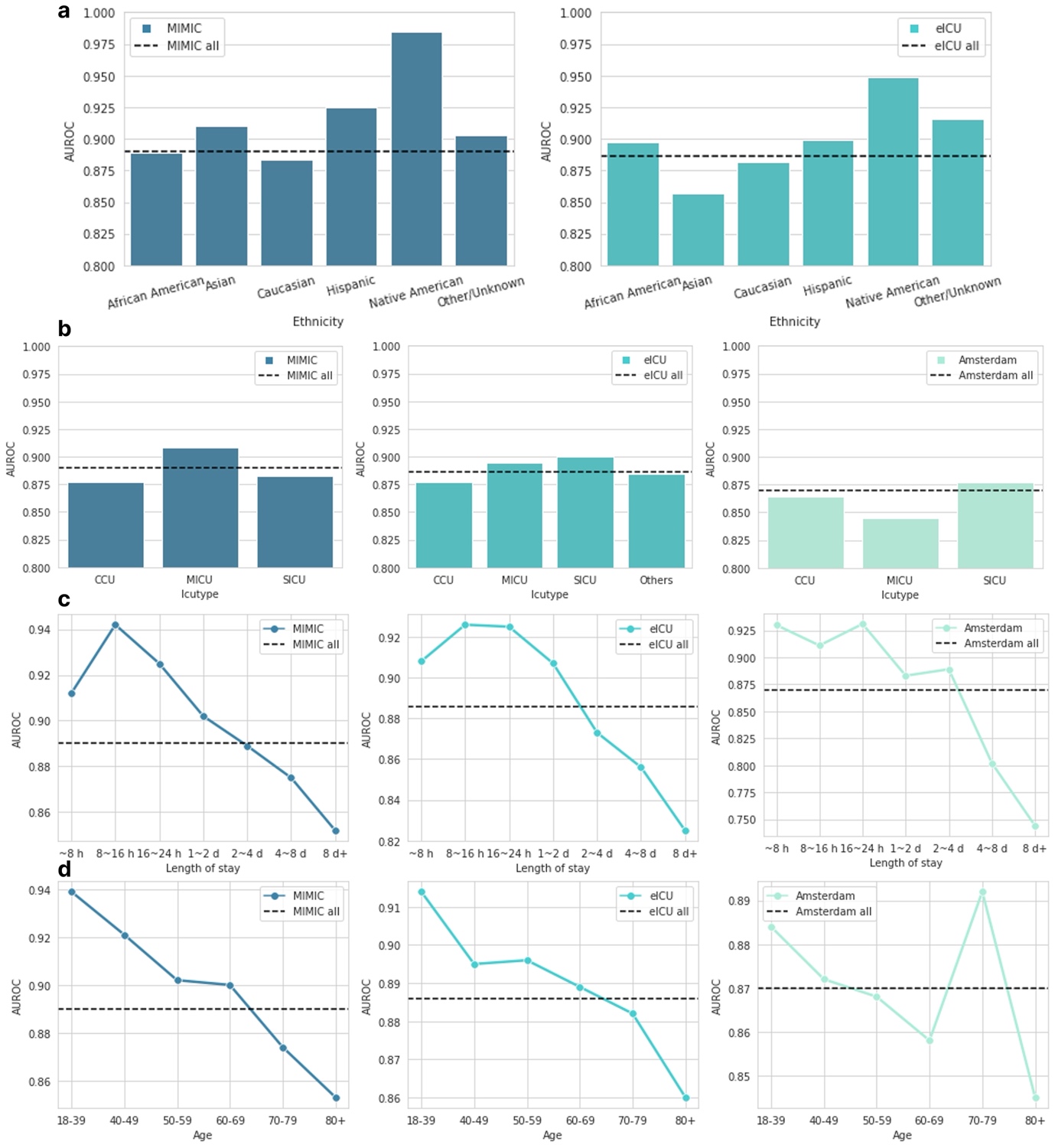


The black dashed line indicates the performance of the entire cohort data.

**a** The icutypes have been grouped into four categories: CCU, MICU, SICU, and Others. Refer to Fig. E8 for a detailed icutype classification. **b** Ethnicities have been classified into six different types. **c** length of stay has been categorized into seven types, considering the admission distribution across the total cohorts. **d** age has been segmented into intervals of 10 years except 18-39.

AUROC=area under the receiver operating characteristic, MIMIC=Mart for Intensive Care, eICU=eICU Collaborative Research Database, Amsterdam=Amsterdam University Medical Center database, CCU=coronary care unit, MICU=medical intensive care unit, SICU=surgical intensive care unit.

**Fig. E10.** Subgroup analysis results.


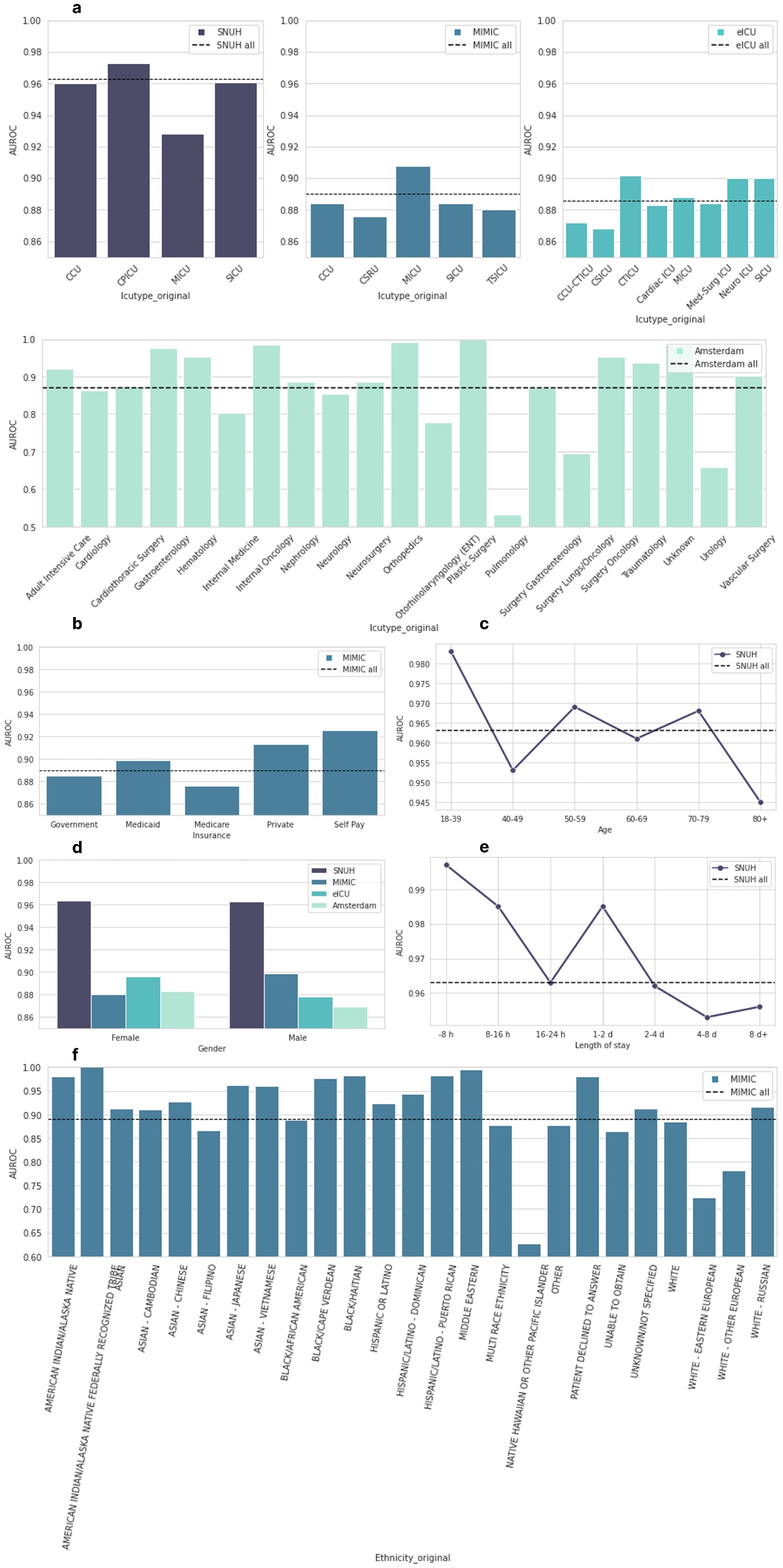


**a** Performance by ICU type or medical specialty for all cohorts. **b** Performance by insurance for MIMIC. **c** performance by age for SNUH. **d** performance by gender for all cohorts. **e** performance by length of ICU stay for SNUH. **f** performance by ethnicity for MIMIC. . *Different icutypes are categorized as follows: For MIMIC, CCU encompasses CCU and CSRU. MICU comprises MICU. SICU includes SICU and TSICU. For eICU, CCU encompasses CCU-CTICU, CSICU, CTICU, and Cardiac ICU. MICU comprises MICU and Neuro ICU. SICU includes SICU. Others consist of Med-Surg ICU. For Amsterdam, ICU types categorized by medical specialty (Table E3).

AUROC=area under the receiver operating characteristic, SNUH=Seoul National University Hospital, MIMIC=Mart for Intensive Care, eICU=eICU Collaborative Research Database, Amsterdam=Amsterdam University Medical Center database, CCU=coronary care unit, CPICU=cardiopulmonary intensive care unit, MICU=medical intensive care unit. SICU=surgical intensive care unit, CSRU=cardiac surgery recovery unit. TSICU=trauma surgical intensive care unit, CCU-CTICU=coronary care unit/cardiothoracic ICU, CSICU=cardiac surgery intensive care unit, CTICU=cardiothoracic intensive care unit, Med-Surg ICU=Medical-surgical intensive care unit, Neuro ICU=Neurological intensive care unit.
